# LncRNA APCDD1L-AS1 induces icotinib resistance by inhibition of EGFR autophagic degradation via the miR-1322/miR-1972/miR-324-3p-SIRT5 axis in lung adenocarcinoma

**DOI:** 10.1186/s40364-021-00262-3

**Published:** 2021-01-30

**Authors:** Jie Wu, Chunlei Zheng, Yizhe Wang, Zichang Yang, Ce Li, Wanxia Fang, Yue Jin, Kezuo Hou, Yang Cheng, Jianfei Qi, Xiujuan Qu, Yunpeng Liu, Xiaofang Che, Xuejun Hu

**Affiliations:** 1 Department of Respiratory and Infectious Disease of Geriatrics, The First Hospital of China Medical University, No.155 Nanjing North Street, Heping District, Shenyang, 110001 Liaoning China; 2 Department of Oncology, The First Affiliated Hospital of Jinzhou Medical University, Jinzhou, 121000 Liaoning China; 3 Department of Medical Oncology, The First Hospital of China Medical University, No.155, North Nanjing Street, Heping District, Shenyang, 110001 Liaoning China; 4 Key Laboratory of Anticancer Drugs and Biotherapy of Liaoning Province, The First Hospital of China Medical University, Shenyang, 110001 Liaoning China; 5 Liaoning Province Clinical Research Center for Cancer, Shenyang, 110001 Liaoning China; 6 Marlene and Stewart Greenebaum Comprehensive Cancer Center, University of Maryland, Baltimore, MD USA

**Keywords:** LncRNA APCDD1L-AS1, Lung adenocarcinoma, Icotinib-resistance, Autophagy, SIRT5

## Abstract

**Background:**

Epidermal growth factor receptor-tyrosinase kinase inhibitor (EGFR-TKI) resistance is the major obstacle in the treatment of lung adenocarcinoma (LUAD) patients harboring EGFR-sensitive mutations. However, the long non-coding RNAs (lncRNAs) related to EGFR-TKIs resistance and their functional mechanisms are still largely unknown. This study aimed to investigate the role and regulatory mechanism of lncRNA APCDD1L-AS1 in icotinib resistance of lung cancer.

**Methods:**

Molecular approaches including qRT-PCR, MTT assay, colony formation, RNA interference and cell transfection, RNA immunoprecipitation (RIP), dual luciferase reporter assay, RNA fluorescence in situ hybridization, TUNEL assay, flow cytometry, immunoblotting, xenograft model and transcriptome sequencing were used to investigate the mechanism of APCDD1L-AS1 in icotinib resistance.

**Results:**

A novel lncRNA, APCDD1L-AS1 was identified as the most significantly upregulated lncRNA in icotinib-resistant LUAD cells by the transcriptome sequencing and differential lncRNA expression analysis. We found that APCDD1L-AS1 not only promoted icotinib resistance, but also upregulated the protein expression level of EGFR. Mechanistically, APCDD1L-AS1 promoted icotinib resistance and EGFR upregulation by sponging with miR-1322/miR-1972/miR-324-3p to remove the transcription inhibition of SIRT5. Furthermore, SIRT5 elevated EGFR expression and activation by inhibiting the autophagic degradation of EGFR, finally promoting icotinib resistance. Consistently, the autophagy initiator rapamycin could decrease EGFR levels and increase the sensitivity of icotinib-resistant LUAD cells to icotinib.

**Conclusion:**

APCDD1L-AS1 could promote icotinib resistance by inhibiting autophagic degradation of EGFR via the miR-1322/miR-1972/miR-324-3p-SIRT5 axis. The combination of autophagy initiator and EGFR-TKIs might serve as a potential new strategy for overcoming EGFR-TKIs resistance in LUAD patients.

**Supplementary Information:**

The online version contains supplementary material available at 10.1186/s40364-021-00262-3.

## Background

Lung cancer is one of the most common malignancies and the leading cause of cancer-related deaths worldwide [1, 2]. In the past ten years, the development of targeted therapy has greatly improved the survival of lung cancer patients, especially those with lung adenocarcinoma (LUAD). Approximately 40% of LUAD patients in Asian carry the sensitive mutations in epidermal growth factor receptor (*EGFR*) [3]. EGFR-TKIs, such as gefitinib, erlotinib and icotinib, have been recommended worldwide as a standard first-line regimen. However, since the majority of the patients acquired drug resistance after initial response to EGFR-TKIs within 10–16 months [4], EGFR-TKIs resistance has become a major obstacle in the treatment of LUAD. The EGFR (T790M) mutation and gene amplification of MET are known to be the main molecular mechanisms of resistance to EGFR-TKIs, whereas the third-generation EGFR-TKIs (Osimertinib) or the combination of MET inhibitors (crizotinib) are wildly used for the therapy of these patients [5]. We and others have reported that the PI3K-AKT-mTOR or STAT3 signaling pathway also contributes to EGFR-TKIs resistance, and the mTOR or STAT3 inhibition could partially reverse this phenomenon [6–9]. In addition, we recently reported that Grb2 was highly expressed in icotinib-resistant LUAD cells, whereas and the combination of Grb2 inhibitor lymecycline could enhance the sensitivity to icotinib [6]. However, a large number of patients still have no effective strategy for overcoming EGFR-TKI resistance [10]. Therefore, it is urgent to further explore the mechanism of EGFR-TKIs resistance, and develop more effective therapeutic strategies in the treatment of LUAD.

With the development of next generation sequencing technologies, it has been well known that only < 2% of the human transcriptional products encode proteins, whereas the remaining 98% are non-coding RNAs (ncRNAs) lacking protein-coding potential [11]. Long non-coding RNAs (lncRNAs) are a novel class of ncRNAs with more than 200 nucleotides in length [12–14]. LncRNAs are known to be involved in various cellular processes such as cell differentiation, autophagy and apoptosis [15]. To date, numerous lncRNAs are recognized as novel biomarkers and therapeutic targets in the diagnosis and treatment of malignancies. Furthermore, mounting studies demonstrated that lncRNAs play a pivotal role in conferring anticancer drug resistance [16, 17]. LncRNA GSTM3TV2 could promote gemcitabine resistance by sponging let-7 with concomitant upregulation of LAT2 and OLR1 in pancreatic cancer [18]; LncRNAH19 induced autophagy and this could contribute to tamoxifen resistance via H19/SAHH/DNMT3B axis in breast cancer [19]; MALAT1 contributed to gefitinib resistance by sponging miR-200a and enhancing ZEB1 expression [20]. However, the studies about EGFR-TKI resistance related lncRNAs are still limited, and their functional mechanisms remain largely unknown.

In the current study, we identified a novel icotinib resistance-related lncRNA, APCDD1L-AS1, and found that APCDD1L-AS1 could upregulateSIRT5 expression by sponging with miR-1322/miR-1972/miR-324-3pin LUAD cells. SIRT5 inhibited autophagic degradation of EGFR to induce icotinib resistance. An autophagy initiator could perfectly reverse icotinib resistance in LUAD cells. Therefore, this study revealed a novel lncRNA-mediated mechanism of icotinib resistance, and provided a potential strategy for overcoming icotinib resistance in LUAD patients.

## Methods

### Establishment of icotinib-resistant cells and cell culture

Human lung adenocarcinoma cell lines (PC9, HCC827) were purchased from American type culture collection (Manassas, VA, USA). The icotinib (Betta Pharmaceuticals Co., Ltd., China) resistant lung adenocarcinoma cells PC9/IcoRL (low-dose icotinib-resistant), PC9/IcoRH (high-dose icotinib-resistant), HCC827/IcoRL and HCC827/IcoRH, without T790M mutation and MET amplification confirmed by sequencing, were established with a gradient concentration of icotinib (0.01–20 μM) for over 6 months. All the cells were maintained in RPMI-1640 medium (Gibco, Waltham, MA, USA) supplemented with 10% fetal bovine serum (HyClone, GE Healthcare LifeSciences, Logan, UT, USA) and were cultured in a humidified incubator at 37 °C with 5% CO_2_. The autophagy inhibitor chloroquine (CQ) and 3-Methyladenine (3-MA) were purchased from Sigma-Aldrich, St. Louis, MO, USA and Selleck Chemicals, Houston, TX, USA, respectively. The autophagy initiator, rapamycin, was purchased from Sigma-Aldrich, USA. The protein synthesis inhibitor cycloheximide (CHX) and the proteasome inhibitor MG-132were purchased from Sigma-Aldrich, USA.

### RNA extraction and qRT-PCR

Total RNA was purified using Trizol reagent (Invitrogen, Carlsbad, CA, USA). PARIS™ Kit (Invitrogen, AM1921) was utilized to isolate nuclear and cytoplasmic RNA according to the manufacturer’s instructions. RNA was reverse-transcribed to cDNA by the PrimeScript™ RT reagent kit (Takara, Tokyo, Japan) and the One Step PrimeScript® miRNAcDNA Synthesis Kit’s (TaKaRa, Japan) following the protocols. Quantitative real-time PCR was performed with the SYBR Premix ExTaq II kit (TaKaRa, Japan) and the Applied Biosystems 7500 Fluorescent Quantitative PCR system (Applied BiosystemsLife Technologies, Carlsbad, CA, USA). The 2^−ΔΔCt^ method was used to calculate the fold change of the relative mRNA expression. U6 or 18S were used as an internal control for normalization. The primer sequences were provided in the supplemental material (Additional file 1: Table S1).

### RNA interference and cell transfection

Specific small interfering RNAs, target to APCDD1L-AS1, SIRT5 and negative control, mimics and inhibitors for miR-1322, miR-1972 and miR-324-3p were synthesized by Ribobio (Guangzhou, China). APCDD1L-AS1-overexpression plasmid (pcDNA3.1-APCDD1L-AS1) and the lentivirus vector containing the APCDD1L-AS1 shRNA (Lv-shRNA-APCDD1L-AS1) were constructed by OBiotech (Shanghai, China). Lv-shRNA-APCDD1L-AS1 stable transfection cells were selected by puromycin (4 μg/ml, Sigma-Aldrich, USA). In addition, siRNAs, miRNA mimics and inhibitors, vectors or negative control were transfected by using Jet PRIME (Polyplus transfection, Strasbourg, France) according to the manufacturer’s instructions. The RNA interference sequences are listed in the supplemental material (Additional file 1: Table S1).

### MTT assay for Icotinib-sensitivity and cell viability

The icotinib-resistant LUAD cells or their parental cells with or without transfection were seed into 96-well plates (2–3 × 10^3^ cells/well), and treated with different concentrations of icotinib for 96 h, followed by the absorbance (570 nm) assessment using the 3-(4, 5-dimethyl-2-thiazolyl)-2, 5-diphenyl-2-H-tetrazolium bromide (MTT, Sigma-Aldrich, USA). Then the 50% inhibition of growth (IC50) value for icotinb was calculated in each cell line as previously described [21]. For cell viability assay, the icotinib-resistant lung adenocarcinoma cells were treated with 10 μM of icotinib for 24 h, 48 h, 72 h and 96 h. The cell viability was also assayed using MTT assay. All experiments were performed in triplicate.

### Colony formation

Cells were seeded into 12-well plate (600 cells/well) in standard medium. Next day, different concentrations of icotinib were added into the medium. Two weeks later, live cells were stained using crystal violet (Sigma-Aldrich, USA), and the number of colonies in each well was counted.

### Xenograft studies

The icotinib-resistant or parental lung adenocarcionma cells (150 μl, approximately 1.0 × 10^7^ cells) were injected subcutaneously into female nude mice (4-week old, Beijing Vital River Laboratory Animal Technology Co., Ltd., China). Two weeks later, the tumor-bearing mice were orally treated with icotinib (50 mg/kg) in 1% Tween-80 saline solution every three days, and the tumor volumes were assessed (0.5 × length×width^2^) per week for 3 consecutive weeks. After all mice were euthanized, the tumors were surgically removed and photographed.

To explore the function of APCDD1L-AS1 in vivo, the icotinib-resistant lung adenocarcinoma cells (150 μl, approximately 1.0 × 10^7^ cells) with stable Lv-sh RNA-APCDD1L-AS1 or Lv-NC were injected subcutaneously into mice. The dosage of icotinib, treatment method and duration were same as above. Partial tumor tissues were immediately frozen in liquid nitrogen for RNA extraction, and others were fixed in 10% buffered formalin for immunohistochemistry, immunofluorescence and Tunel assay. The antibodies for immunohistochemical staining were EGFR (1:100, Santa, Clara, CA, USA), SIRT5 (1:100, Sigma-Aldrich, USA). The antibody for immunofluorescence staining was LC3B (1:200, Cell Signaling Technology, Danvers, MA, USA). The images were quantified using the default ‘Analyze particles’ plugin in image analyser (Image Pro Plus 6.0, Media Cybernetics), and results were presented in average LC3B puncta numbers per each cell according to the guidelines of autophagy [22]. Tunel staining was performed on paraffin section using one-step tunnel apoptosis assay kit (Byotime, Jiangsu, China) according to the manufacturer’s instructions. The image analyser (Image Pro Plus 6.0, Media Cybernetics) was used to score all the staining sections of Tunel, and results were presented in mean gray value [23]. This study was performed in animal laboratory center of China Medical University and was granted by the Institutional Review Board of China Medical University (IACUC Issue No: 2019067).

### RNA immunoprecipitation (RIP)

The procedure was performed using Magna RNA-binding protein immunoprecipitation kit (Millipore, Billerica, MA, USA) as per the manufacturer’s protocol. Human anti-AGO2 antibody (Abcam, Cambridge, USA) and anti-rabbit IgG (Millipore, USA) were used for RIP. The immunoprecipitated RNAs were isolated and detected by qRT-PCR. The gene-specific primer sequences used for the qRT-PCR of the RIP assay were listed in the supplemental material (Additional file 1: Table S1).

### Dual luciferase reporter assay

Luciferase reporter plasmids of wild type- and mutant-APCDD1L-AS1 (Luc-APCDD1L-AS1-wt and Luc-APCDD1L-AS1-mut) and SIRT5 (Luc-SIRT5-wt and Luc-SIRT5-mut) were individually constructed by OBiotech (Shanghai, China). HEK-293 cells in 96-well plate (4000 cells per well) were co-transfected with plasmids of synthesized dual-luciferase reporters and miRNA mimics for 48 h, and the dual-luciferase activity were detected using the Dual-Luciferase Reporter Assay System Kit (Promega, Madison, WI, USA) according to the manufacturer’s instructions. Firefly luciferase activity was normalized to renilla luciferase activity.

### RNA fluorescence in situ hybridization

Cy3-labeled APCDD1L-AS1 was purchased from ServiceBio (Wuhan, China). RNA FISH were performed as described previously using Fluorescent in Situ Hybridization kit (ServiceBio, China) following the manufacturer’s instructions. The slides were observed for immunofluorescence with a fluorescence microscope (Eclipse Ti-SR, Nikon). U6 and 18S probes (ServiceBio, China) were used as internal reference of nuclear and cytoplasmic RNA.

### Flow cytometry for the detection of apoptosis

Icotinib-resistant LUAD cells were treated with icotinib (5 μM or 10 μM) for 72 h. Then both floating and attached cells were harvested, and staining with Annexin V-FITC Apoptosis Detection kit. Cells apoptosis were analyzed by FACS cytometry (BD Biosciences Inc., Franklin, NJ, USA).

### Western blot

Western blot was carried out as previously described [24]. These following primary antibodies were used: EGFR, PARP, LC3B and p62 (1:1000, Cell Signaling Technology, USA), p-EGFR (1:250, Cell Signaling Technology, USA), SIRT5 (1:1000, Sigma-Aldrich, USA) and GAPDH (1:5000, Cell Signaling Technology, USA).

### Statistical analysis

The data were shown as mean ± standard deviation (SD). The paired Student’s t test or One-Way ANOVA was used to compare the differences among two or multiple groups. A two-tailed *P* < 0.05 was deemed to be statistically significant while *P* < 0.01 was very significant. All data analyses were carried out with GraphPad Prism software 5.0 (GraphPad Software, Inc., San Diego, CA, USA) and SPSS 16.0 (IBM, SPSS, Chicago, IL, USA).

## Results

### APCDD1L-AS1 was significantly up-regulated in icotinib-resistant LUAD cells

**Fig. 1:**
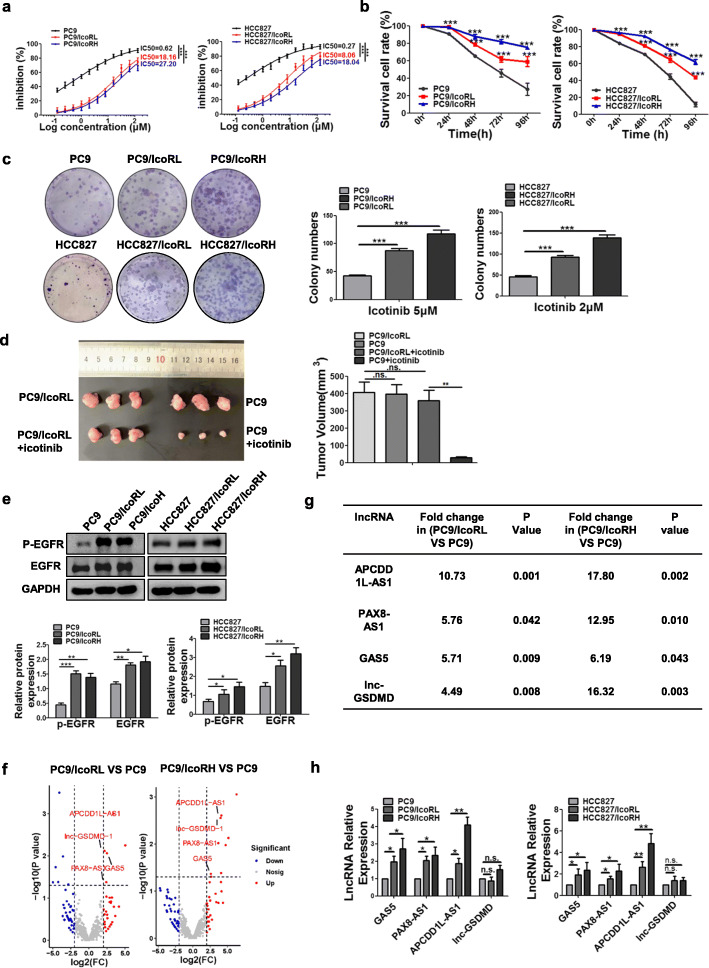
Significant upregulation of APCDD1L-AS1 in icotinib-resistant LUAD cells. **a** The icotinib sensitivity in icotinib-resistant LUAD cells and their parental cells treated with different concentrations of icotinib for 96 h was determined by MTT assay. PC9/IcoRL: PC9 low-dose icotinib-resistant cells; PC9/IcoRH: PC9 high-dose icotinib-resistant cells; HCC827/IcoRL: HCC827 low-dose icotinib-resistant cells; HCC827/IcoRH: HCC827 high-dose icotinib-resistant cells. **b** The cell viability of both the parental cells and their icotinib-resistant cells after treated with icotinib (10 μM) for 24, 48, 72 and 96 h was detected by MTT assay. **c** The colony formation ability of the parental cells and their icotinib-resistant cells under different concentrations of icotinib was analyzed using colony formation assay. **d** The subcutaneous tumor mouse models of icotinib-resistant cells and their parental cells were treated with or without icotinib. Average tumor volume for each group was measured (*n* = 3). **e** The level of EGFR expression and phosphorylation in the parental cells and their icotinib-resistant cells was evaluated by western blot. **f** Four upregulated lncRNAs identified by volcano plots in PC9/IcoRL cells and PC9/IcoRH cells comparing with PC9 cells. **g** The list of top four upregulated lncRNAs in PC9/IcoRL cells and PC9/IcoRH cells comparing with PC9 cells by transcriptome sequencing. **h** The expression level of lncRNAs, APCDD1L-AS1, PAX8-AS1, GAS5 and lnc-GSDMD, was determined in the parental cells and their icotinib-resistant cells by qRT-PCR. The mean ± SD of triplicate experiments were plotted, *P < 0.05, **P < 0.01, ***P < 0.001, n.s., not statistically significant

Firstly, the icotinib sensitivity was compared in parental (PC9 and HCC827) and icotinib-resistant cell lines (PC9/IcoRL, PC9/IcoRH, HCC827/IcoRL and HCC827/IcoRH). MTT assays showed that icotinib sensitivity was much lower inicotinib-resistant cells than their parental cells in a dose- and time-dependent manner (Fig. 1a-b). The colony formation assay also showed that the colonies of the parental cells were significantly smaller and less than those of icotinib-resistant cells after icotinib treatment (Fig. 1c). Similarly, subcutaneous xenograft tumors of PC9 cell group showed smaller size than those of PC9/IcoRL cell group after icotinib treatment (Fig. 1d). Additionally, the protein and the phosphorylation levels of EGFR were significantly higher in icotinib-resistant cells than parental cells (Fig. 1e). All these results confirmed the icotinib-resistant phenotype in the LUAD cells used in our study.

To screen the lncRNAs related to icotinib resistance, we performed the global expression profiles analysis of lncRNA in PC9, PC9/IcoRL and PC9/IcoRH cells. Among the differentially expressed lncRNAs, APCDD1L-AS1 was the most up-regulated lncRNA in both PC9/IcoRL and PC9/IcoRH cells compared to PC9 cells (Fig. 1f-g). Similar results were also verified by qRT-PCR (Fig. 1h). Due to the significant upregulation of APCDD1L-AS1, we decided to further investigate its potential role in icotinib resistance.

### APCDD1L-AS1 contributed to the icotinib resistance of LUAD cells

**Fig. 2:**
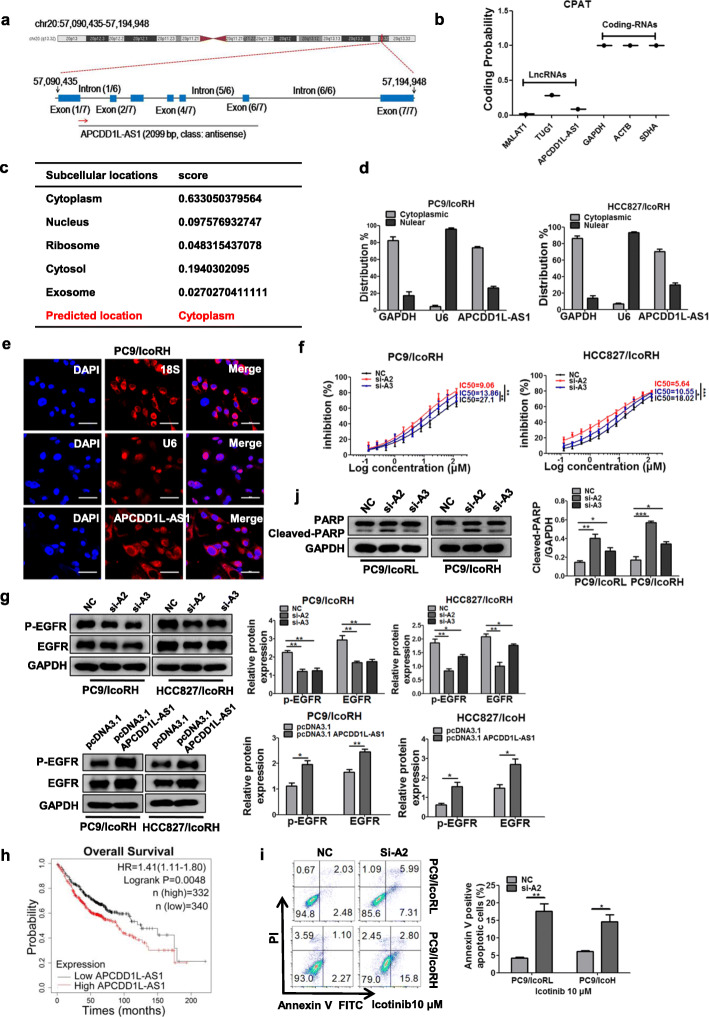
Contribution of APCDD1L-AS1 to the icotinib resistance of LUAD cells. **a** The localization and structure of APCDD1L-AS1 on the chromosome in the LNCipedia database. **b** The coding potentials of lncRNAs (MALAT1, TUG1, APCDD1L-AS1) and mRNAs (GAPDH, ACTB, SDHA) were calculated using CPAT database. **c** The online software lncLocator was used to predict the subcellular localization of APCDD1L-AS1. **d** Relative expression of APCDD1L-AS1 in cytoplasm or nucleus of the icotinib-resistant cells (PC9/IcoRH, HCC827/IcoRH) was determined by qRT-PCR. **e** The localization of APCDD1-AS1 in the PC9/IcoRH cells was detected by RNA-FISH. Blue, DAPI-stained nuclei; Red, Cy3-labeled positive hybridization signals (scale bar, 100 μm). U6 and 18S were used as positive controls. **f** The effect of APCDD1L-AS1 KD on the IC50 value of icotinib was evaluated in icotinib-resistant cells (PC9/IcoRH, HCC827/IcoRH) by MTT assay. **g** The level of EGFR and p-EGFR in APCDD1L-AS1-KD or -OE icotinib-resistant cells (PC9/IcoRH, HCC827/IcoRH) was determined by western blot. **h** Kaplan-Meier analyses of the correlations between APCDD1L-AS1 expression (classified into high and low expression groups according to the median of APCDD1L-AS1 expression) and OS in 672 lung adenocarcinoma patients using Kaplan-Meier Plotter online database. Log rank test was used to calculate *P* values. **i-j** The apoptosis of APCDD1L-AS1-KD icotinib-resistant cells (PC9/IcoRL, PC9/IcoRH) induced by icotinib (10 μM) was analyzed using flow cytometry (**i**), and apoptosis-related protein PARP in PC9/IcoRL and PC9/IcoRH cells was detected by western blot (**j**)**.** GAPDH was used as the internal control. The mean ± SD of triplicate experiments were plotted, *P < 0.05, **P < 0.01, ***P < 0.001

APCDD1L-AS1, located at chromosomal 20q13.32 and composed of seven exons with a full length of 2099-nucleotide (nt) (Fig. 2a), has been identified as an lncRNA in the LNCipedia database (version 5.2). The prediction result of Coding Potential Assessment Tool further confirmed that APCDD1L-AS1 only possessed a very low coding potential (Fig. 2b). To explore the subcellular localization of APCDD1L-AS1, we searched the database lncLocator and the results predicted the predominant cytoplasmic localization of APCDD1L-AS1 with minor nuclear localization (Fig. 2c). This result was further verified by the nuclear/cytoplasmic RNA separation analysis using qRT-PCR and RNA fluorescence in situ hybridization (FISH) assay in icotinib-resistant LUAD cells (Fig. 2d-e; Additional file 1: Figure S1A and S1B).

To determine whether APCDD1L-AS1was involved in icotinib resistance, we transiently knocked down or overexpressed APCDD1L-AS1 in icotinib-resistant cells (Additional file 1: Figure S1C and S1D). The result showed that APCDD1L-AS1 knock down (KD) could significantly enhance the icotinib sensitivity (Fig. 2f; Additional file 1: Figure S1E) and decrease the protein and phosphorylation levels of EGFR (Fig. 2g; Additional file 1: Figure S1F). In contrast, APCDD1L-AS1 overexpression (OE) increased the expression and activation of EGFR (Fig. 2g; Additional file 1: Figure S1F). The similar results were also obtained with gefitinib treatment (Additional file 1: Figure S1G). Kaplan-Meier survival analysis of 672 LUAD patients showed that high expression of APCDD1L-AS1 was significantly associated with worse overall survival (OS) (log rank test, *p* = 0.0048; Fig. 2h). Additionally, flow cytometry and western blot analyses confirmed that APCDD1L-AS1KD promoted icotinib-induced apoptosis in icotinib-resistant cells (Fig. 2i-j; Additional file 1: Figure S1H and S1I). Collectively, these results supported a role for APCDD1L-AS1 in EGFR upregulation and icotinitib resistance.

### APCDD1L-AS1 sponged miR-1322/miR-1972 /miR-324-3p to induceicotinib resistance of LUAD cells

**Fig. 3:**
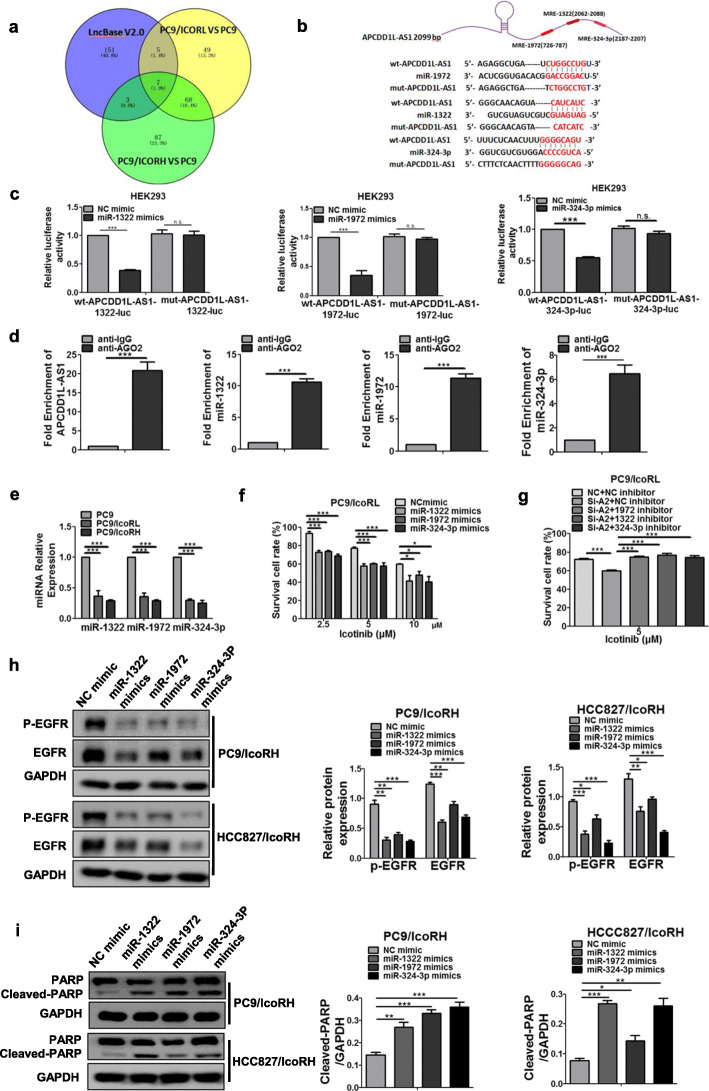
MiR-1322/miR-1972/miR-324-3p were sponged by APCDD1L-AS1 and improved the sensitivity of LUAD cells to icotinib. **a** Potential miRNAs (miR-1322, miR-1972 and miR-324-3p) sponging with APCDD1L-AS1 were predicted by LncBasedatabase and transcriptome sequencing. **b** Sequence alignment of wild type and mutant type of APCDD1L-AS1 with miR-1322, miR-1972 and miR-324-3p potential targeting sites. **c** Luciferase reporter assay using miRNA mimics was applied to verify the interaction between APCDD1L-AS1 and miR-1322, miR-1972 or miR-324-3p. **d** RIP assay was performed using AGO2 antibody, immunoprecipitation of APCDD1L-AS1 and miR-1322, miR-1972 or miR-324-3p were determined by qRT-PCR. IgG was used as a negative control. **e** The expression level of miRNAs in the parental cells and their icotinib-resistant cells (PC9, PC9/IcoRL and PC9/IcoRH) was determined by qRT-PCR. **f** The effect of miR-1322, miR-1972 or miR-324-3p mimics on the icotinib sensitivity of PC9/IcoRL cells was determined by MTT assay. **g** After co-transfection with miR-1322, miR-1972 or miR-324-3p inhibitor and si-RNA APCDD1L-AS1, the viability of PC9/IcoRL cells with the treatment of icotinib for 72 h was determined by MTT assay. **h-i** The level of EGFR, p-EGFR (**h**) and PARP (**i**) in the icotinib-resistant cells (PC9/IcoRH and HCC827/IcoRH) with the transfection of miR-1322, miR-1972 or miR-324-3p mimics was determined by western blot. The mean ± SD of triplicate experiments were plotted, *P < 0.05, **P < 0.01, ***P < 0.001, n.s., not statistically significant

LncRNAs localized in cytoplasm usually exert their regulatory functions by sponging with miRNAs, which is known as the competing endogenous RNA (ceRNA) [25]. To predict the potential miRNAs sponged with APCDD1L-AS1, we used the LncBase V2.0 [26] and identified three miRNAs (miR-1972, miR-1322 and miR-324-3p) with higher binding scores (Fig. 3a-b). The putative binding sites of these miRNAs are at positions 726 to 787, 2062 to 2088, 2187 to 2207, respectively (Fig. 3b). Then dual luciferase reporter assays were carried out by co-transfecting the plasmid of wild type- or mutant putative binding sites APCDD1L-AS1 (Luc-APCDD1L-AS1-wt or Luc-APCDD1L-AS1-mut) and miR-1972/miR-1322/miR-324-3p mimics into HEK293 cells. The results showed that all three miRNA mimics could suppress luciferase activity driven by APCDD1L-AS1-wt, but not by APCDD1L-AS1-mut at the presumptive miRNAs binding sites (Fig. 3c). Furthermore, RIP assays showed that Argonaute 2 (Ago2), the essential component of RNA-induced silence complex (RISC), associated with the complex of APCDD1L-AS1, miR-1972, miR-1322 and miR-324-3p (Fig. 3d).

Results of qRT-PCR showed that the levels of all three miRNAs were lower in icotinib-resistant cells compared to parental cells (Fig. 3e; Additional file 1: Figure S2A). In contrast, the levels of all these miRNAs were significantly increased in APCDD1L-AS1-KD cells, while decreased in APCDD1L-AS1-OE cells (Additional file 1: Figure S2B and S2C; Figure S3A and S3B). On the other hand, the mimics of miR-1322, miR-1972 and miR-324-3p decreased, whereas their inhibitors increased the level of APCDD1L-AS1 (Additional file 1: Figure S3C). All these results indicated that APCDD1L-AS1 could sponge with miR-1322, miR-1972 and miR-324-3p in a reciprocally suppressive manner.

To investigate whether miR-1322, miR-1972 and miR-324-3p were involved in icotinib resistance, the mimics or inhibitors of miR-1322, miR-1972 or miR-324-3p were separately transfected into icotinib-resistant LUAD cells. The result showed that all the mimics significantly enhanced icotinib sensitivity (Fig. 3f; Additional file 1: Figure S4A), whereas three inhibitors all partially attenuated the enhanced icotinib sensitivity observed in the APCDD1L-AS1-KD cells (Fig. 3g; Additional file 1: Figure S4B). Further, the mimics of all three miRNAs not only alleviated the protein and phosphorylation levels of EGFR, but also promoted apoptosis in icotinib-resistant cells (Fig. 3h-i). Collectively, these data indicated that APCDD1L-AS1 sponged with miR-1322/miR-1972/miR-324-3p, contributing to EGFR upregulation and icotinib resistance.

### MiR-1322/miR-1972/miR-324-3p down-regulated SIRT5 by targeting its 3′-UTRs

**Fig. 4:**
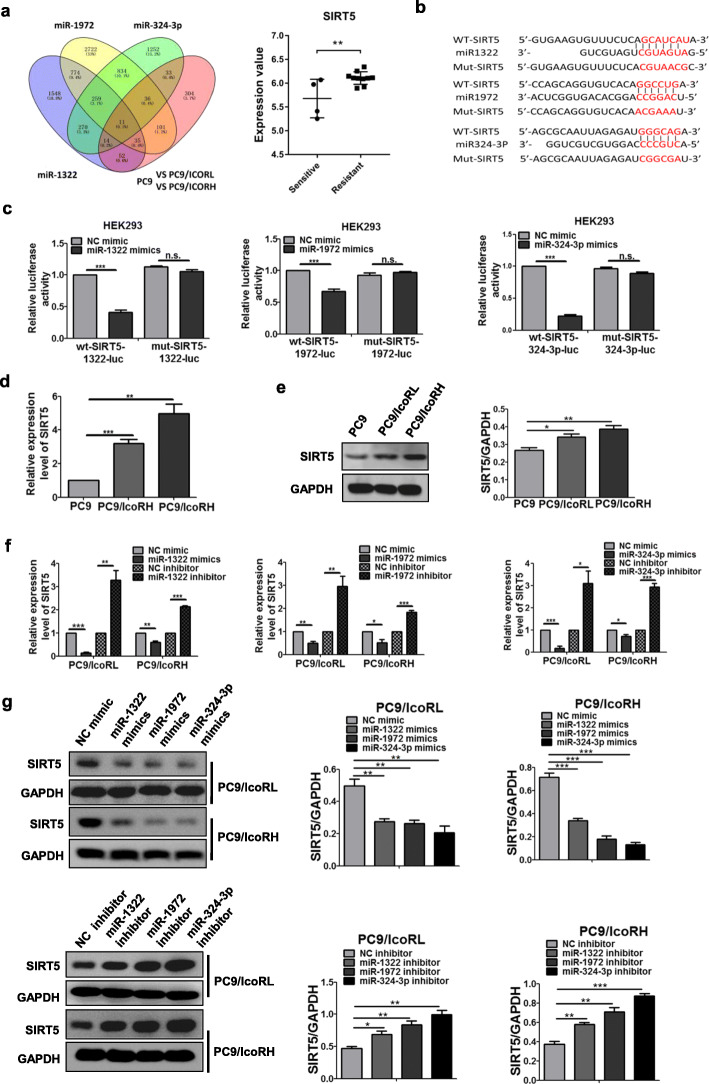
Downregulation of SIRT5 by miR-1322/miR-1972/miR-324-3p through targeting its 3′-UTRs. **a** The possible target genes of miR-1322, miR-1972 and miR-324-3p were predicted by Targetscan and transcriptome sequencing; the mRNA level of SIRT5 from GEO dataset GSE80344 in gefitinib-resistance cells and their sensitive cells was shown. **b** Potential binding sites of miR-1322, miR-1972 and miR-324-3p in SIRT5 3′-UTR. **c** Luciferase reporter assay using miRNA mimics was applied to verify the interaction between SIRT5 3′-UTR and miR-1322, miR-1972 or miR-324-3p. **d-e** The expression of SIRT5in the parental cells and their icotinib-resistant cells (PC9, PC9/IcoRL and PC9/IcoRH) was detected by qRT-PCR (**d**) and western blot (**e**). **f-g** The SIRT5 expression in PC9/IcoRL and PC9/IcoRH cells with the transfection of miRNAs mimics or inhibitor was detected by qRT-PCR (**f**) and western blot (**g**). The mean ± SD of triplicate experiments were plotted, *P < 0.05, **P < 0.01, ***P < 0.001

To screen common mRNA targets of miR-1322, miR-1972 and miR-324-3p, we performed Targetscan prediction and differentially expressed genes (DEGs) analysis of our sequencing results. Among the 11 predictive mRNAs, sirtuin 5 (SIRT5), a NAD^+^-dependent class III protein deacetylase, showed a significantly higher expression in gefitinib-resistant than sensitive NSCLC samples in the GEO: GSE80344 dataset (Fig. 4a). Therefore, we selected SIRT5 as a possible target inicotinib resistance (Fig. 4a). To test whether SIRT5 was a target of the three miRNAs, the plasmids of Luc-SIRT5 3′-UTR-wt and Luc-SIRT5 3′-UTR-mut were co-transfected with the mimics of three miRNAs (Fig. 4b). Luciferase assays showed that the mimics of all three miRNAs could only repress the luciferase activity of Luc-SIRT5 3′-UTR-wt but not that of Luc-SIRT5 3′-UTR-mut (Fig. 4c), suggesting that all three miRNAs could directly bind to SIRT5 3′-UTRs. Moreover, the mRNA and protein levels of SIRT5 were significantly higher in icotinib-resistant LUAD cells than parental cells (Fig. 4d-e; Additional file 1: Figure S5A and S5B). Further, the levels of SIRT5 were down-regulated by mimics of all three miRNAs, while up-regulated by inhibitors of these miRNAs (Fig. 4f-g; Additional file 1: Figure S5C and S5D). The data above indicated that SIRT5 was a downstream target of APCDD1L-AS1-miR-1322/miR-1972/miR-324-3p axis.

### SIRT5 contributed to icotinib resistance in LUAD cells

**Fig. 5:**
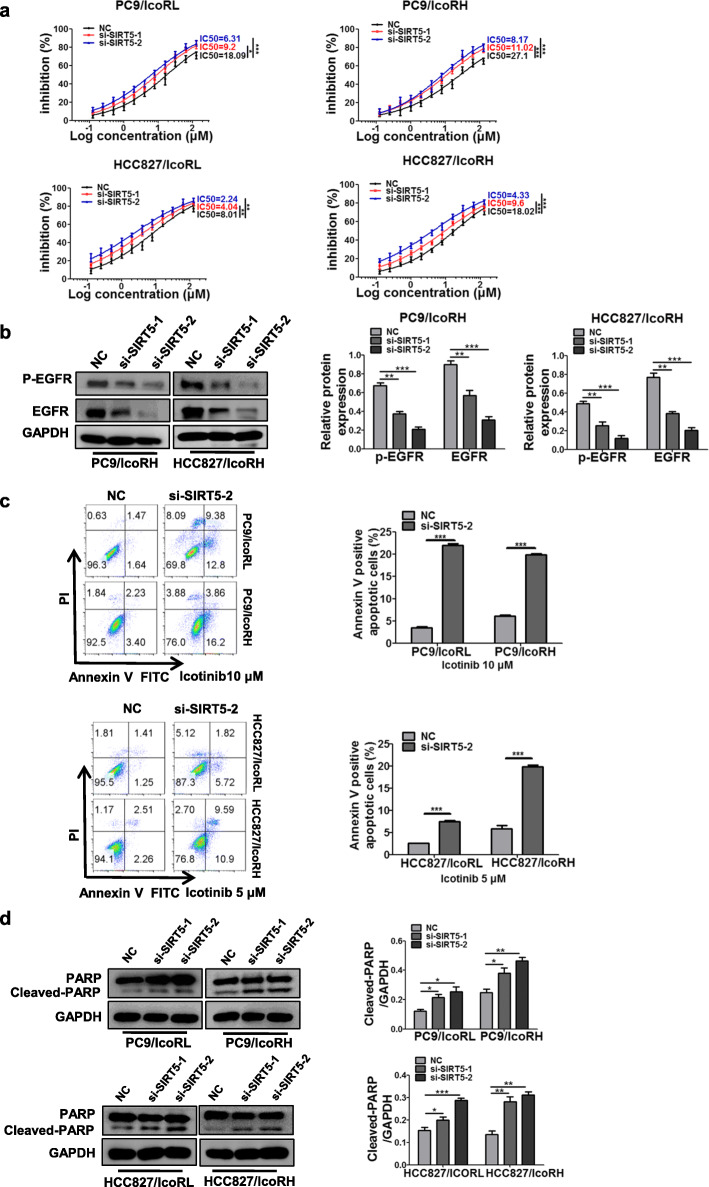
Contribution of SIRT5 to icotinib resistance. **a** The IC50 value of icotinibinSIRT5 KD-icotinib-resistant cells was determined by MTT assay. **b** The level of EGFR and p-EGFR in SIRT5 KD-icotinib-resistant cells (PC9/IcoRH and HCC827/IcoRH) was detected by western blot. **c-d** The apoptosis of SIRT5 KD-icotinib-resistant cells with the treatment of icotinib was analyzed by flow cytometry (**c**), and apoptosis-related protein PARP was assessed by western blot (**d**). The mean ± SD of triplicate experiments were plotted, *P < 0.05, **P < 0.01, ***P < 0.001

To verify the role of SIRT5 in icotinib resistance, SIRT5 was knocked down in icotinib-resistant LUAD cells (Additional file 1: Figure S6A). The MTT assays showed that SIRT5-KD significantly reduced the IC50 values for icotinib in icotinib-resistant cells (Fig. 5a). Meanwhile, both protein and phosphorylation levels of EGFR were significantly decreased in SIRT5-KD icotinib-resistant cells (Fig. 5b; Additional file 1: Figure S6B). In addition, flow cytometry and western blot analyses indicated that SIRT5 KD significantly increased apoptosis in icotinib-resistant cells (Fig. 5c-d). These results indicated that SIRT5 contributed to EGFR upregulation following icotinib resistance.

### SIRT5 up-regulated EGFR by inhibiting autophagic degradation

**Fig. 6:**
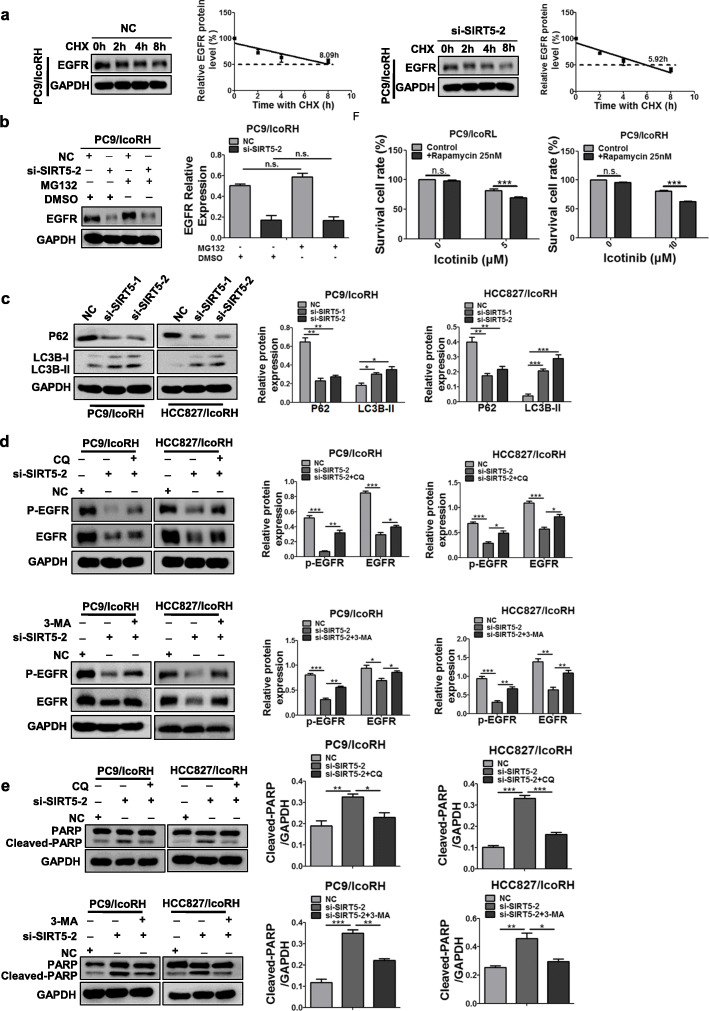
Inhibition of autophagic degradation of EGFR by SIRT5. **a** The protein level of EGFR in PC9/IcoRH cells with the treatment of CHX (20 μg/ml) for 0, 2, 4 and 8 h were determined by western blot. **b** The protein level of EGFR in SIRT5 KD-PC9/IcoRH cells with the treatment of MG-132 (10 μM) for 12 h were determined by western blot. **c** The level of LC3B and p62 in SIRT5 KD-icotinib-resistant cells (PC9/IcoRH and HCC827/IcoRH) was determined by western blot. **d-e** The level of EGFR, p-EGFR (**d**) and PARP (**e**) in SIRT5 KD-icotinib-resistant LUAD cells (PC9/IcoRH and HCC827/IcoRH) treated with CQ or 3-MA was determined by western blot. **f** The icotinib sensitivity in icotinib-resistant LUAD cells (PC9/IcoRL and PC9/IcoRH) with or without rapamycinfor 72 h was determined by MTT assay. The mean ± SD of triplicate experiments were plotted, *P < 0.05, **P < 0.01, ***P < 0.001, n.s., not statistically significant

The next question is how SIRT5 up-regulated EGFR expression. No changes of EGFR mRNA levels was observed in SIRT5-KD cells (Additional file 1: Figure S7A) indicating that SIRT5 might increase EGFR protein synthesis and/or inhibiting EGFR degradation. Then, after treated with the protein synthesis inhibitor cyclohexane (CHX), the protein level of EGFR in SIRT5-KD icotinib-resistant cells was analyzed by western blot. The results showed that SIRT5-KD dramatically shortened the half-life of EGFR (Fig. 6a; Additional file 1: Figure S7B), suggesting that SIRT5 might upregulate EGFR by inhibiting its degradation. However, the proteasome inhibitor MG-132 could not elevate EGFR levels in SIRT5-KD cells, excluding the possible involvement of proteasome pathway in the process (Fig. 6b; Additional file 1: Figure S7C).

Autophagy is another well known pathway for the degradation of damaged proteins. Therefore, we determined to test whether EGFR undergone the autophagic degradation in the SIRT5-KD cells. Our results showed the decreased level of p62, and increased level of LC3B-II in SIRT5-KD cells (Fig. 6c, Additional file 1: Figure S7D), indicating that SIRT5 KD could promote autophagic flux in icotinib-resistant LUAD cells. Furthermore, two autophagy inhibitors, CQ and 3-MA, partially rescued SIRT5-KD-induced phenotypes including EGFR downregulation, decreased EGFR activation and increased PARP cleavage (Fig. 6d-e; Additional file 1: Figure S7E and S8A). On the contrary, the combination of autophagy initiator rapamycin with icotinib partially reversed the icotinib resistance of LUAD cells (Fig. 6f). More importantly, the images of confocal microscopy showed that EGFR partially colocalized with the LC3B autophagic vesicle marker under SIRT5 knockdown in HCC827/IcoRH cells (Additional file 1: Figure S8B). Taken together, these results suggested that SIRT5 promoted icotinib resistance by inhibiting EGFR autophagic degradation.

### APCDD1L-AS1 up-regulated SIRT5 by sponging miR1322/miR1972/miR324-3p

**Fig. 7:**
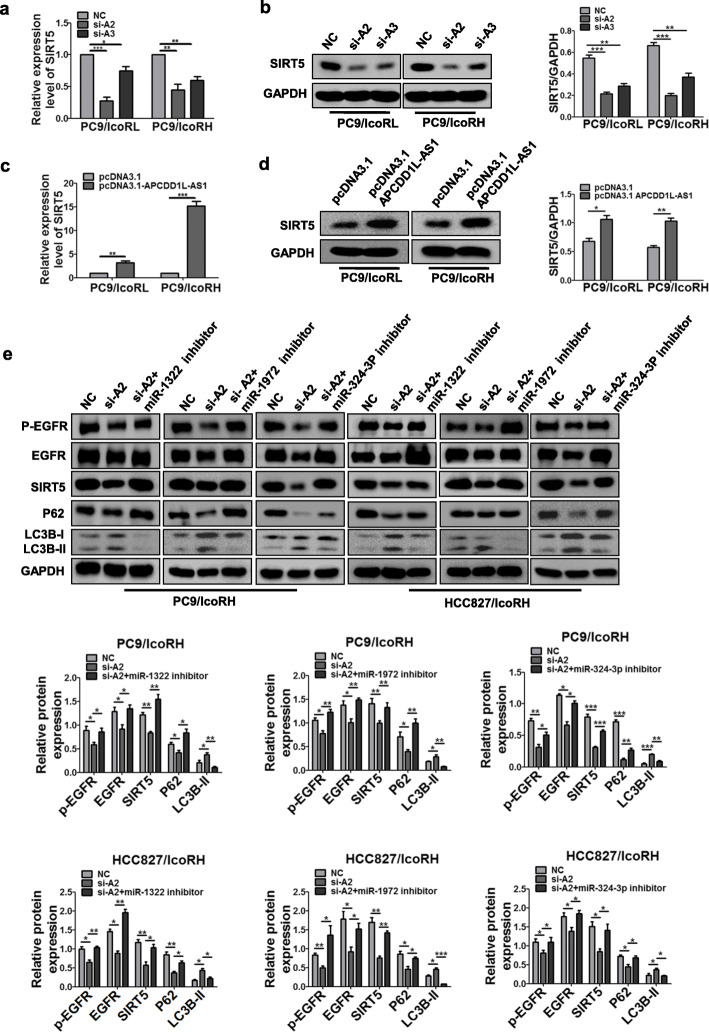
Upregulation of SIRT5 by APCDD1L-AS1 through sponging with miR-1322/miR-1972/miR-324-3p. **a-d** The RNA and protein expression of SIRT5 were detected in PC9/IcoRL and PC9/IcoRH cells with APCDD1L-AS1KD (**a-b**) or OE (**c-d**) by qRT-PCR and western blot. **e** The level of p-EGFR, EGFR, SIRT5, p62 and LC3B in APCDD1L-AS1 KD-icotinib-resistant LUAD cells co-transfected with the inhibitors of miR-1322, miR-1972 and miR-324-3p was determined by western blot, respectively. The mean ± SD of triplicate experiments were plotted, *P < 0.05, **P < 0.01, ***P < 0.001

To further confirm whether SIRT5-supressing EGFR degradation is regulated by ceRNA network of APCDD1L-AS1 and miR1322/miR1972/miR324-3p, the effect of APCDD1L-AS1 on the expression of SIRT5 and EGFR was examined. The results showed that both the mRNA and protein levels of SIRT5 were decreased by APCDD1L-AS1-KD, but increased by APCDD1L-AS1-OE (Fig. 7a-d; Additional file 1: Figure S9A-S9D). Additionally, APCDD1L-AS1-KD also inhibited EGFR expression and its activation, as well as promoted the autophagic flux, which were partially rescued by inhibitors of miR-1322, miR-1972 and miR-324-3p (Fig. 7e). Taken together, these results indicated that the APCDD1L-AS1-miR-1322/miR-1972/ miR-324-3p-SIRT5 axis promoted icotinib-resistance by inhibiting autophagic degradation of EGFR.

### APCDD1L-AS1 enhanced icotinib resistance by inhibiting autophagic degradation of EGFR in xenograft mouse model

**Fig. 8:**
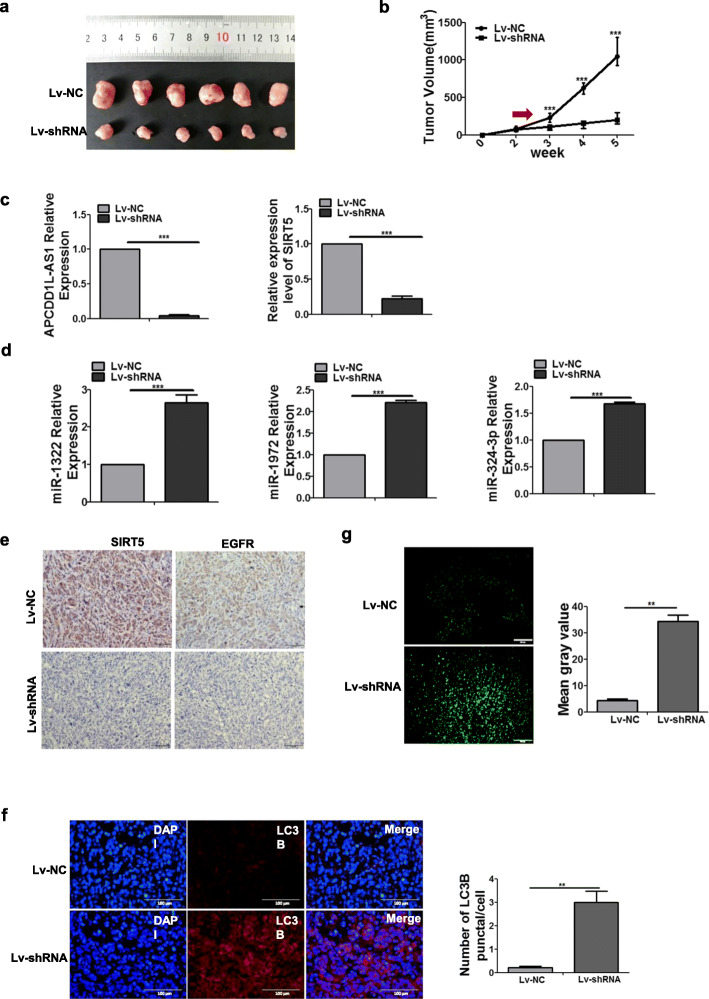
Enhancement of icotinib sensitivity by APCDD1L-AS1 knockdown through promoting autophagic degradation of EGFR in xenograft mouse model. **a-b** The tumors isolated from nude mice subcutaneously injected with PC9/IcoRH/Lv-sh-APCDD1L-AS1 or PC9/IcoRH/Lv-NC cells were shown (**a**) (*n* = 6). Tumor growth curves were drawn according to the size detected per week (**b**). **c-d** The expression of APCDD1L-AS1, SIRT5 (**c**), miR-1322, miR-1972 and miR-324-3p (**d**) was analyzed by qRT-PCR assay. **e** The expression of SIRT5 and EGFR was determined by immunohistochemical staining (scale bar, 50 μm). **f** The LC3B punctures were observed by immunofluorescence (scale bar, 100 μm). The nuclei were visualized using DAPI. Representative images are presented on the figure. **g** The cell apoptosis of tumor sections was determined by TUNEL assay (scale bar, 100 μm). The mean gray value represented the status of cell apoptosis (Lv-NC group = 4.39 ± 0.568, Lv-shRNA group = 34.39 ± 2.273). The mean ± SD of triplicate experiments were plotted, **P < 0.01, ***P < 0.001

**Fig. 9:**
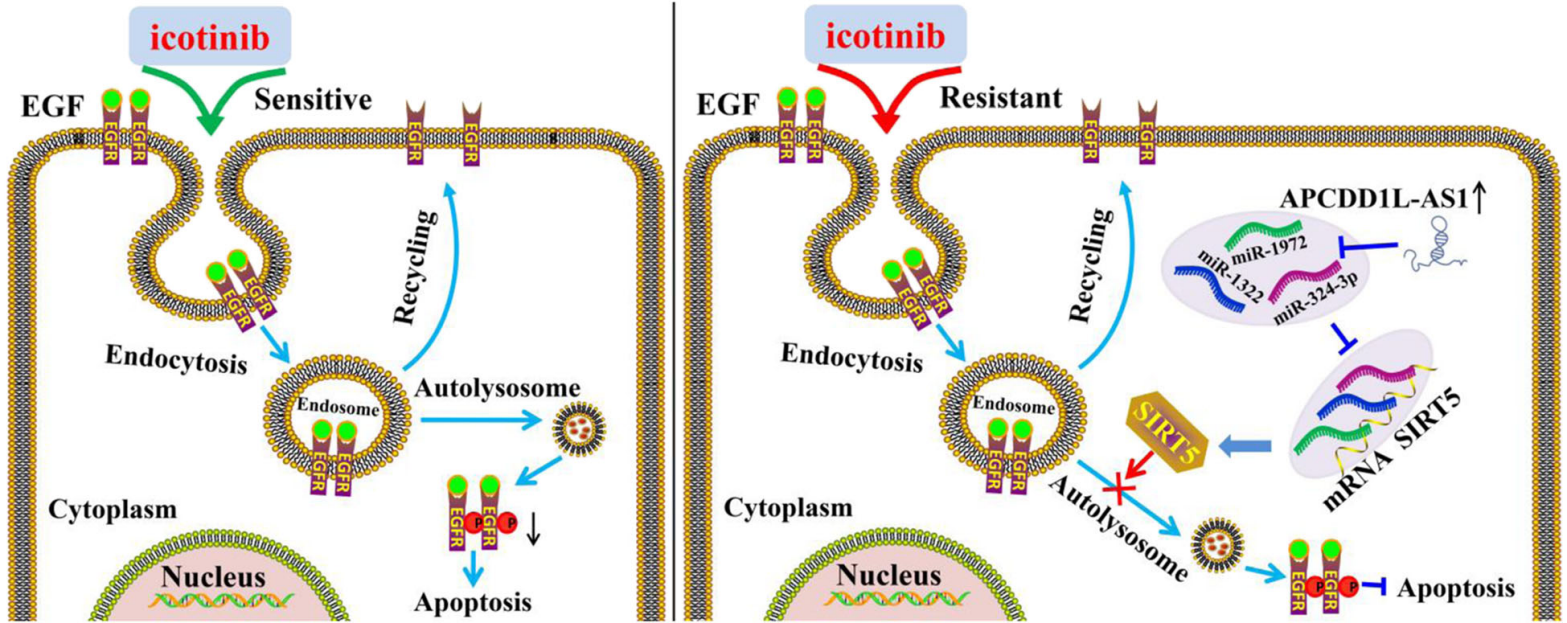
The schematic diagram of APCDD1L-AS1 contributing to icotinib resistance in lung adenocarcinoma cells. In this progress, up-regulated APCDD1L-AS1 as a miRNA sponge to decoy miR-1322, miR-1972 and miR-324-3p, promote the expression of SIRT5, inhibit autophagic degradation of EGFR, increase EGFR phosphorylation, inhibit apoptosis and induce icotinib resistance

To further verify the function and mechanisms of APCDD1L-AS1 on icotinib resistance in vivo, we stably transfected PC9/IcoRH cells with lentiviral vectors (Lv-shRNA-APCDD1L-AS1 or Lv-NC) for the xenograft tumor model study. With icotinib treatment, tumors in the Lv-shRNA-APCDD1L-AS1 group were significantly smaller than those in Lv-NC group (Fig. 8a-b). Furthermore, qRT-PCR detection confirmed that the expressions of APCDD1L-AS1 and SIRT5 were lower, and the expressions of miR-1322, miR-1972 and miR-324-3p were significantly higher in Lv-shRNA-APCDD1L-AS1 tumor tissues than the Lv-NC tumor tissues (Fig. 8c-d). Immuno-histochemical staining showed that the levels of SIRT5 and EGFR were dramatically decreased in the Lv-shRNA-APCDD1L-AS1 tumors (Fig. 8e). Meanwhile, immunofluorescence staining showed that both LC3B puncta and tunnel positive staining were substantially increased in the Lv-shRNA-APCDD1L-AS1 tumors (Fig. 8f-g). These data were consistent with our in vitro results, further supporting our model that APCDD1L-AS1 contributed to icotinib resistance by inhibiting autophagic degradation of EGFR via the miR-1322/miR-1972/miR-324-3p-SIRT5 axis (Fig. 9).

## Discussion

In the present study, we found the upregulation of a novel lncRNA APCDD1L-AS1 in icotinib-resistant LUAD cells, and proposed a novel working model wherein APCDD1L-AS1 induced icotinib resistance by inhibiting autophagic degradation of EGFR and promoted EGFR activation in LUAD through the miR-1322/miR-1972/miR-324-3p-SIRT5 axis.

Aberrant expressions of numerous lncRNAs have been found in various cancers, including lung cancer. The ceRNA network composed of lncRNAs-miRNA-mRNA is one of the main mechanisms promoting cancer progression. A single lncRNA can interact with single or multiple miRNAs, and a single miRNA can also interact with single or multiple lncRNAs or mRNAs. Therefore, the ceRNA network is highly complex [27]. Studies concerning EGFR-TKI resistance in NSCLC reported that lncRNA LOC554202 upregulating miR-31 could reduce sensitivity of NSCLC cells to gefitinib [28]. The lncRNA RHPN1-AS1 downregulation promoted gefitinib resistance by targeting the miR-299-3p/TNFSF12 pathway in NSCLC [29]. However, these studies just focused on individual ceRNA interactions. In this study, based on the prediction of target miRNAs and mRNAs and the confirmation of luciferase reporter assay and RIP, we found that APCDD1L-AS1 could simultaneously sponge with three miRNAs (miR-1972, miR-1322 and miR-324-3p), and the three miRNAs targeted the common gene, SIRT5, establishing a stable and powerful ceRNA network. As a result, APCDD1L-AS1 exerted strong drug resistance-promoting function through this ceRNA network.

We found that SIRT5 could promote EGFR-TKI resistance. SIRT5, a member of Sirtuin family, is known to play important roles in autophagy, apoptosis and drug resistance [30, 31], A study based on online analysis indicated that SIRT5 was positively correlated with complete response to neoadjuvant chemotherapy in triple negative breast cancer, suggesting that SIRT5 might play an inhibitory role in drug resistance [32]. However, it was also reported that SIRT5-positive colorectal cancer cells with wild-type KRAS were resistant to either chemotherapeutic agents or cetuximab [33]; SIRT5 promoted cisplatin resistance in ovarian cancer. In this study, we found the upregulation of SIRT5 in icotinib-resistant LUAD cells and gefitinib-resistant tumors from GSE80344 dataset, supporting that the resistance promoting role of SIRT5. Also, the findings that SIRT5-KD could promote autophagic degradation of EGFR and enhance icotinib sensitivity provided further evidence that SIRT5 functioned as a resistant factor by inhibiting autophagy. SIRT5 has been reported to regulate autophagy in a context-dependent manner. SIRT5 could promote autophagy in CRC [30], and suppress autophagy in human breast cancer cells [34]. Our results showed that SIRT5 functioned as an autophagy inhibitor, and were consistent with a previous study showing that SIRT5 inhibited autophagy by decreasing ammonia production [34]. Therefore, SIRT5 functioned as an autophagy suppressor in the context of EGFR-TKI resistance.

Autophagy is a conserved catabolic process resulting in self-digestion and the removal of dysfunctional proteins and organelles [35]. Accumulating evidence supported that autophagy, as a double-edged sword, could either induce cell death or cell protection, and might therefore be associated with drug resistance of cancer cells [36]. It was reported that the knockdown of Rab5a or CCL2 could stimulate autophagy to reverse cisplatin resistance in gastric cancer [37]. Our results that the autophagy inhibitors, CQ and 3-MA, rescued SIRT5-KD-induced EGFR autophagic degradation and promoted icotinib resistance, strongly suggested the effect of SIRT5 on icotinib resistance is mediated by its autophagy inhibitory function. Targeting the WEE1 kinase was reported to strengthen the antitumor activity of imatinib via promoting KIT autophagic degradation in gastrointestinal stromal tumors [38]. In our study, we found that knockdown of SIRT5 could decrease EGFR expression and increased the colocalization of EGFR with autophagic vesicles. Also, SIRT5 knockdown promoted autophagic degradation of EGFR, suggesting that the LncRNA-miR-1322/miR-1972/miR-324-3p-SIRT5 axis inhibited the autophagic degradation of EGFR. As the key target of EGFR-TKI in NSCLC, EGFR was reported to be up-regulated at both protein and phosphorylation levels in EGFR-TKIs resistant cells [39], which was similar to our results. Therefore, accelerating autophagic degradation of EGFR may be the potential strategy for overcoming EGFR-TKI resistance in LUAD. In the current study, mTOR inhibitor rapamycin, which can also induce cell death via promoting autophagy [40], actually did increase icotinib sensitivity in icotinib-resistant LUAD cells. Other mTOR inhibitors, such as torin2 and BIBW2992) [8, 9] were also reported to be able to induce apoptosis and inhibit cell proliferation in EGFR-TKI-resistant NSCLC cells by negative feedback regulation of Akt/mTOR signaling and inducing autophagy, suggesting promising therapeutic strategy in NSCLC with EGFR-TKI resistant phenotype. In addition, a recent study reported that the combination of bisdemethoxy curcumin and icotinib could enhance the sensitivity of primary EGFR-TKI resistant NSCLC cell lines to icotinib via autophagy induction [41], which was similar to ours. Therefore, our study provided strong evidence that manipulating the activity of autophagy might be a useful therapeutic strategy to enhance the drug sensitivity in cancer [42, 43]. Certainly, due to the complexity of autophagy, the roles of autophagy in icotinib resistance need to be further clarified. Also, it needs to be further addressed whether rapamycin could reverse EGFR-TKI resistance by modulating lncRNA-miR-1322/miR-1972/ miR-324-3p-SIRT5 axis-mediated autophagy in clinical application in the future.

## Conclusions

In summary, this study demonstrated that APCDD1L-AS1 could induce icotinib resistance by inhibiting autophagic degradation of EGFR via a ceRNA network of simultaneously decoying miR-1322, miR-1972 and miR-324-3p to upregulate SIRT5 in LUAD. These findings revealed a novel mechanism of EGFR-TKI resistance, and provided a series of new targets and potential strategy for overcoming EGFR-TKI resistance in LUAD.

## Supplementary Information

**Additional file 1: Figure S1**. EGFR up-regulation and apoptosis inhibition by APCDD1L-AS1 in icotinib-resistant LUAD cells. **Figure S2**. Efficiency of miR-1322/miR-1972/miR-324-3p knockdown and overexpression. **Figure S3**. Reciprocal suppression by APCDD1L-AS1 sponging with miR-1322, miR-1972 and miR-324-3p. **Figure S4**. Reversal of icotinib resistance by miR1322/miR1972/miR324-3p in LUAD cells. **Figure S5**. Negative regulation of SIRT5 by miR-1322/miR-1972/miR-324-3p. **Figure S6**. EGFR down-regulation after SIRT5 knockdown in icotinib-resistant LUAD cells. **Figure S7**. Acceleration of EGFR degradation by SIRT5 knockdown. **Figure S8**. EGFR colocalized with the autophagic vesicle marker LC3B during SIRT5 knockdown. **Figure S9**. Negative regulation of SIRT5 by APCDD1L-AS1. **Table S1.** Information of the qRT-PCR primer, siRNA and shRNA sequence.

## Data Availability

Data and materials will be shared.

## Abbreviations

EGFR: Epidermal growth factor receptor
TKI: tyrosinase kinase inhibitor
LUAD: lung adenocarcinoma
lncRNA: long non-coding RNA
RIP: RNA immunoprecipitation
PI3K: Phosphoinositide 3-kinase
mTOR: the mammalian target of rapamycin
STAT3: Signal transducer and activator of transcription 3
ncRNA: non-coding RNA
FISH: RNA fluorescence in situ hybridization
KD: knock down
OE: overexpression
ceRNA: competing endogenous RNA
Ago2: Argonaute 2
UTR: untranslated region
SIRT5: Sirtuin 5
CHX: cyclohexane
