# Correction to: LncRNA APCDD1L-AS1 induces icotinib resistance by inhibition of EGFR autophagic degradation via the miR-1322/miR-1972/ miR-324-3p-SIRT5 axis in lung adenocarcinoma

**DOI:** 10.1186/s40364-021-00279-8

**Published:** 2021-04-14

**Authors:** Jie Wu, Chunlei Zheng, Yizhe Wang, Zichang Yang, Ce Li, Wanxia Fang, Yue Jin, Kezuo Hou, Yang Cheng, Jianfei Qi, Xiujuan Qu, Yunpeng Liu, Xiaofang Che, Xuejun Hu

**Affiliations:** 1 Department of Respiratory and Infectious Disease of Geriatrics, The First Hospital of China Medical University, No.155 Nanjing North Street, Heping District, Shenyang, 110001 Liaoning China; 2 Department of Oncology, The First Affiliated Hospital of Jinzhou Medical University, Jinzhou, 121000 Liaoning China; 3 Department of Medical Oncology, The First Hospital of China Medical University, No.155, North Nanjing Street, Heping District, Shenyang, 110001 Liaoning China; 4 Key Laboratory of Anticancer Drugs and Biotherapy of Liaoning Province, The First Hospital of China Medical University, Shenyang, 110001 Liaoning China; 5 Liaoning Province Clinical Research Center for Cancer, Shenyang, 110001 Liaoning China; 6 Marlene and Stewart Greenebaum Comprehensive Cancer Center, University of Maryland, Baltimore, MD USA

**Correction to: Biomark Res 9, 9 (2021)**

**https://doi.org/10.1186/s40364-021-00262-3**

The original article [1] contained an error in Corresponding Authorship presentation which has since been corrected.
