# Correction: LncRNA APCDD1L-AS1 induces icotinib resistance by inhibition of EGFR autophagic degradation via the miR-1322/miR-1972/ miR-324-3p-SIRT5 axis in lung adenocarcinoma

**DOI:** 10.1186/s40364-023-00496-3

**Published:** 2023-05-12

**Authors:** Jie Wu, Chunlei Zheng, Yizhe Wang, Zichang Yang, Ce Li, Wanxia Fang, Yue Jin, Kezuo Hou, Yang Cheng, Jianfei Qi, Xiujuan Qu, Yunpeng Liu, Xiaofang Che, Xuejun Hu

**Affiliations:** 1 Department of Respiratory and Infectious Disease of Geriatrics, The First Hospital of China Medical University, No.155 Nanjing North Street, Heping District, Shenyang, 110001 Liaoning China; 2 Department of Oncology, The First Affiliated Hospital of Jinzhou Medical University, Jinzhou, 121000 Liaoning China; 3 Department of Medical Oncology, The First Hospital of China Medical University, No.155, North Nanjing Street, Heping District, Shenyang, 110001 Liaoning China; 4 Key Laboratory of Anticancer Drugs and Biotherapy of Liaoning Province, The First Hospital of China Medical University, Shenyang, 110001 Liaoning China; 5 Liaoning Province Clinical Research Center for Cancer, Shenyang, 110001 Liaoning China; 6 Marlene and Stewart Greenebaum Comprehensive Cancer Center, University of Maryland, Baltimore, MD USA

**Correction: Biomark Res 9, 9 (2021)**

**https://doi.org/10.1186/s40364-021-00262-3**

In the original article [1], there was a mistake in Fig. 1e as published. The pictures of GAPDH of PC9 and HCC827 cells in Fig. 1e were misused by accident during figure assembly. The corrected Fig. 1e appears below with new histograms.

**Fig. 1:**
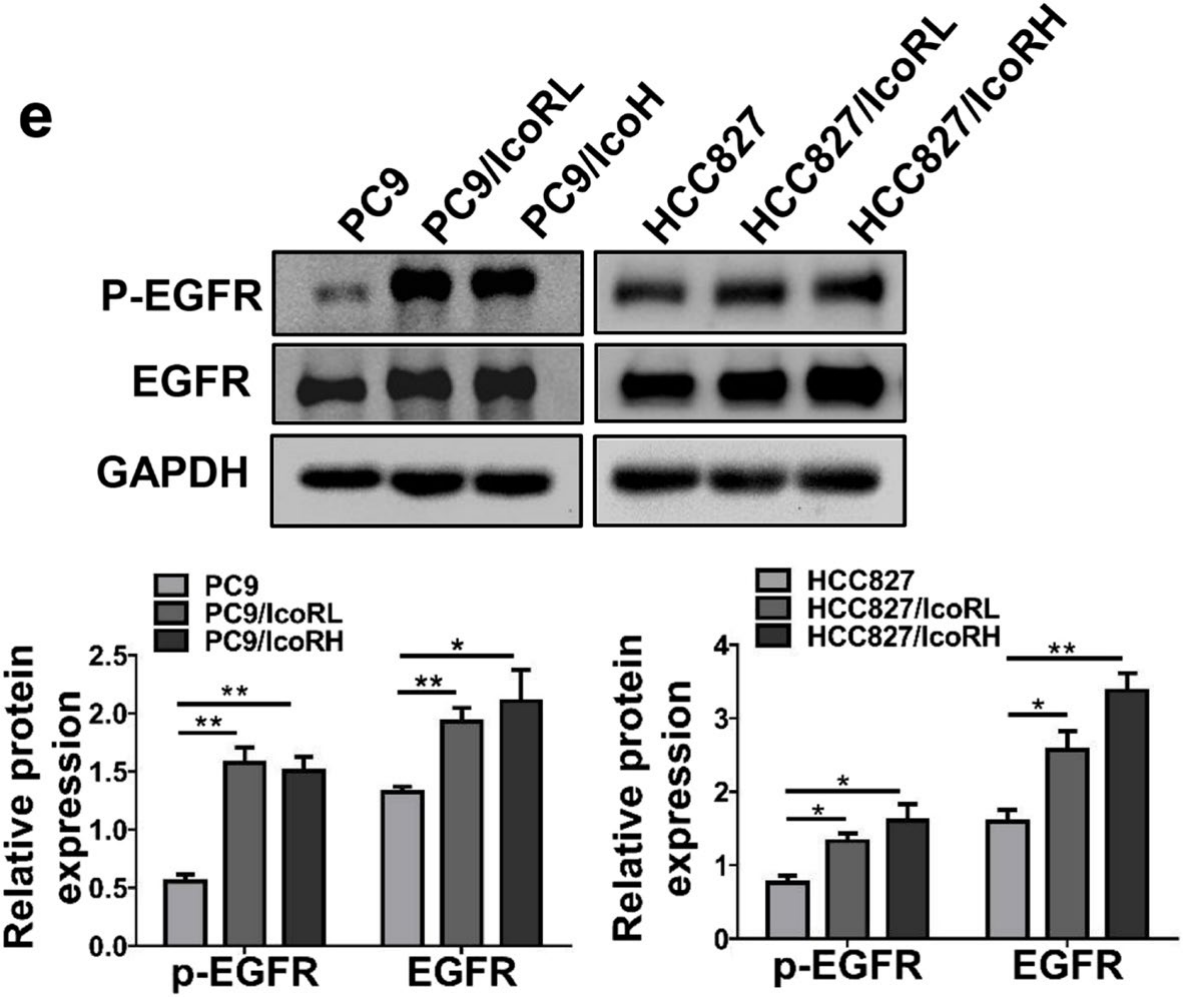
Significant upregulation of APCDD1L-AS1 in icotinib-resistant LUAD cells. **a** The icotinib sensitivity in icotinib-resistant LUAD cells and their parental cells treated with different concentrations of icotinib for 96 h was determined by MTT assay. PC9/IcoRL: PC9 low-dose icotinib-resistant cells; PC9/IcoRH: PC9 high-dose icotinib-resistant cells; HCC827/IcoRL: HCC827 low-dose icotinib-resistant cells; HCC827/IcoRH: HCC827 high-dose icotinib-resistant cells. **b** The cell viability of both the parental cells and their icotinib-resistant cells after treated with icotinib (10 μM) for 24, 48, 72 and 96 h was detected by MTT assay. **c** The colony formation ability of the parental cells and their icotinib-resistant cells under different concentrations of icotinib was analyzed using colony formation assay. **d** The subcutaneous tumor mouse models of icotinib-resistant cells and their parental cells were treated with or without icotinib. Average tumor volume for each group was measured (*n* = 3). **e** The level of EGFR expression and phosphorylation in the parental cells and their icotinib-resistant cells was evaluated by western blot. **f** Four upregulated lncRNAs identified by volcano plots in PC9/IcoRL cells and PC9/IcoRH cells comparing with PC9 cells. **g** The list of top four upregulated lncRNAs in PC9/IcoRL cells and PC9/IcoRH cells comparing with PC9 cells by transcriptome sequencing. **h** The expression level of lncRNAs, APCDD1L-AS1, PAX8-AS1, GAS5 and lnc-GSDMD, was determined in the parental cells and their icotinib-resistant cells by qRT-PCR. The mean ± SD of triplicate experiments were plotted, *P < 0.05, **P < 0.01, ***P < 0.001, n.s., not statistically significant

The authors apologize for this error and state that this does not change the scientific conclusions of the article in any way.
