# Supplementary material for: LncRNA APCDD1L-AS1 induces icotinib resistance by inhibition of EGFR autophagic degradation via the miR-1322/miR-1972/miR-324-3p-SIRT5 axis in lung adenocarcinoma
Source: Biomark Res. 2021 Jan 30;9:9. doi: 10.1186/s40364-021-00262-3 (PMC7847171; doi:10.1186/s40364-021-00262-3)

## Slide 1
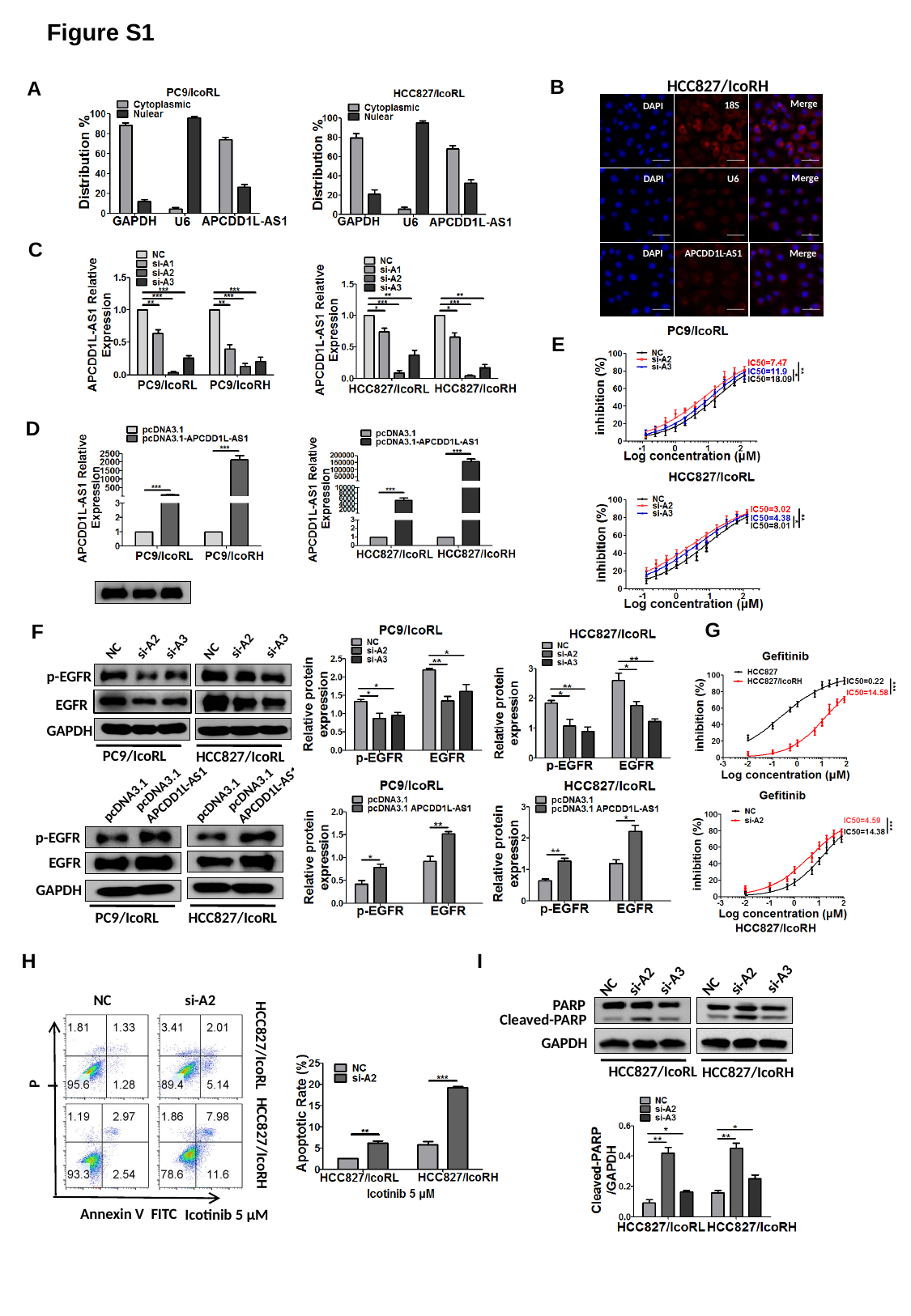

Figure S1
B
HCC827/IcoRH
A
Merge
18S
DAPI
Merge
U6
DAPI
C
APCDD1L-AS1
DAPI
Merge
E
D
si-A2
si-A3
NC
si-A2
si-A3
NC
G
F
p-EGFR
 EGFR
 GAPDH
PC9/IcoRL
HCC827/IcoRL
pcDNA3.1
APCDD1L-AS1
pcDNA3.1
APCDD1L-AS1
pcDNA3.1
pcDNA3.1
p-EGFR
 EGFR
 GAPDH
PC9/IcoRL
HCC827/IcoRL
si-A2
si-A2
NC
 si-A3
NC
 si-A3
H
I
NC
si-A2
PARP
 Cleaved-PARP
GAPDH
HCC827/IcoRL
HCC827/IcoRL
HCC827/IcoRH
PI
HCC827/IcoRH
Annexin V FITC
Icotinib 5 μM

## Slide 2
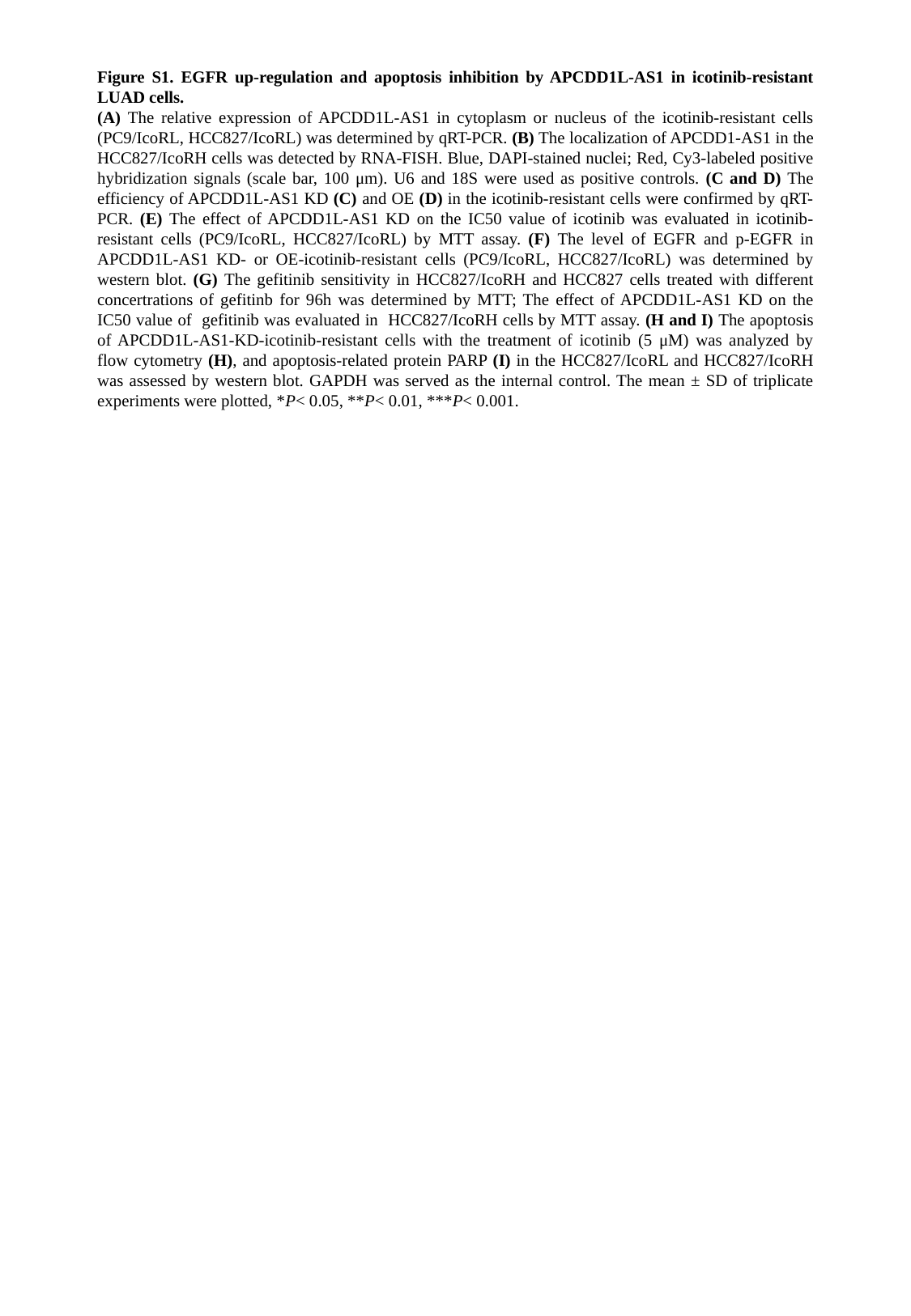

Figure S1. EGFR up-regulation and apoptosis inhibition by APCDD1L-AS1 in icotinib-resistant LUAD cells.
(A) The relative expression of APCDD1L-AS1 in cytoplasm or nucleus of the icotinib-resistant cells (PC9/IcoRL, HCC827/IcoRL) was determined by qRT-PCR. (B) The localization of APCDD1-AS1 in the HCC827/IcoRH cells was detected by RNA-FISH. Blue, DAPI-stained nuclei; Red, Cy3-labeled positive hybridization signals (scale bar, 100 μm). U6 and 18S were used as positive controls. (C and D) The efficiency of APCDD1L-AS1 KD (C) and OE (D) in the icotinib-resistant cells were confirmed by qRT-PCR. (E) The effect of APCDD1L-AS1 KD on the IC50 value of icotinib was evaluated in icotinib-resistant cells (PC9/IcoRL, HCC827/IcoRL) by MTT assay. (F) The level of EGFR and p-EGFR in APCDD1L-AS1 KD- or OE-icotinib-resistant cells (PC9/IcoRL, HCC827/IcoRL) was determined by western blot. (G) The gefitinib sensitivity in HCC827/IcoRH and HCC827 cells treated with different concertrations of gefitinb for 96h was determined by MTT; The effect of APCDD1L-AS1 KD on the IC50 value of gefitinib was evaluated in HCC827/IcoRH cells by MTT assay. (H and I) The apoptosis of APCDD1L-AS1-KD-icotinib-resistant cells with the treatment of icotinib (5 μM) was analyzed by flow cytometry (H), and apoptosis-related protein PARP (I) in the HCC827/IcoRL and HCC827/IcoRH was assessed by western blot. GAPDH was served as the internal control. The mean ± SD of triplicate experiments were plotted, *P< 0.05, **P< 0.01, ***P< 0.001.

## Slide 3
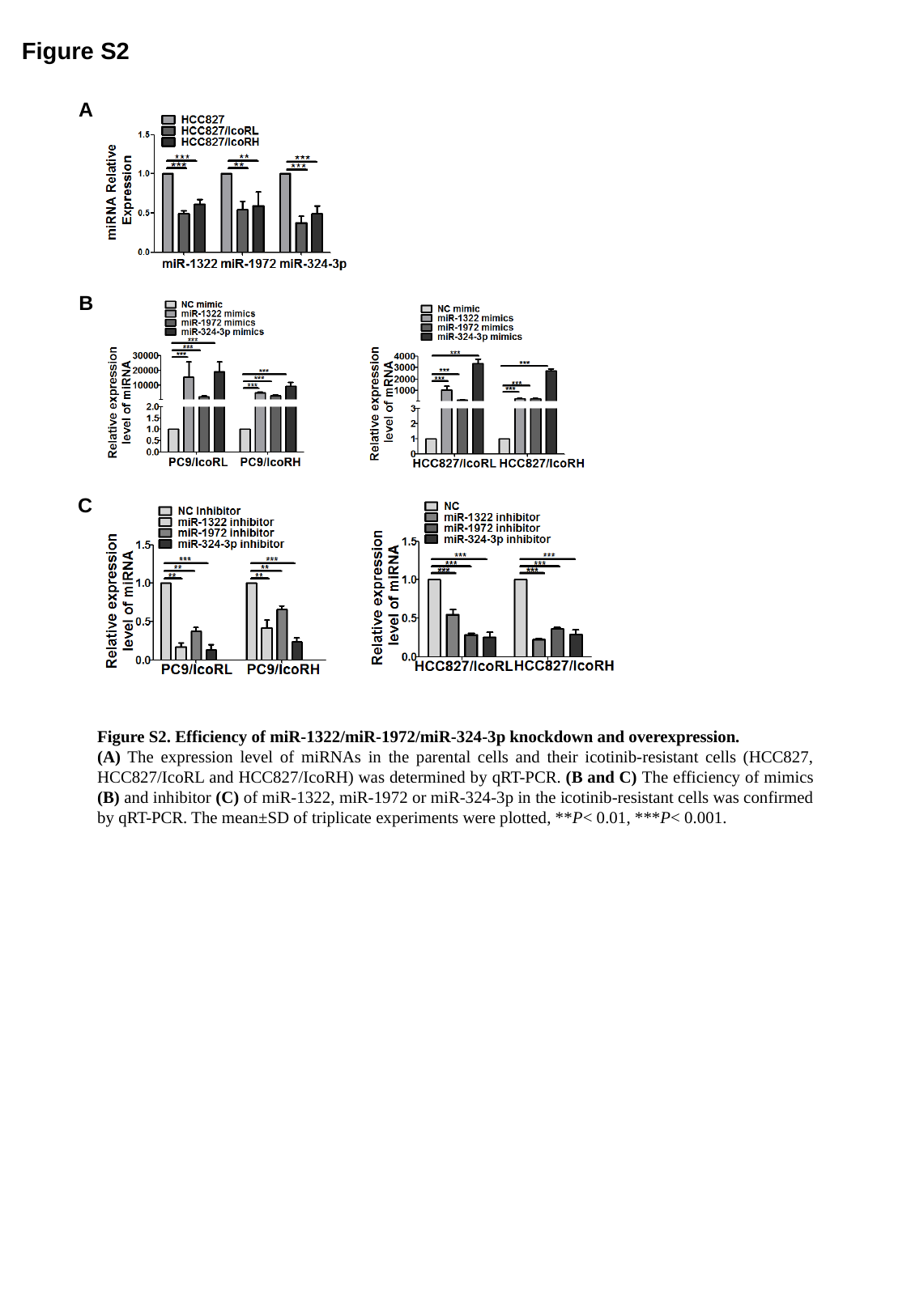

Figure S2
A
B
C
Figure S2. Efficiency of miR-1322/miR-1972/miR-324-3p knockdown and overexpression.
(A) The expression level of miRNAs in the parental cells and their icotinib-resistant cells (HCC827, HCC827/IcoRL and HCC827/IcoRH) was determined by qRT-PCR. (B and C) The efficiency of mimics (B) and inhibitor (C) of miR-1322, miR-1972 or miR-324-3p in the icotinib-resistant cells was confirmed by qRT-PCR. The mean±SD of triplicate experiments were plotted, **P< 0.01, ***P< 0.001.

## Slide 4
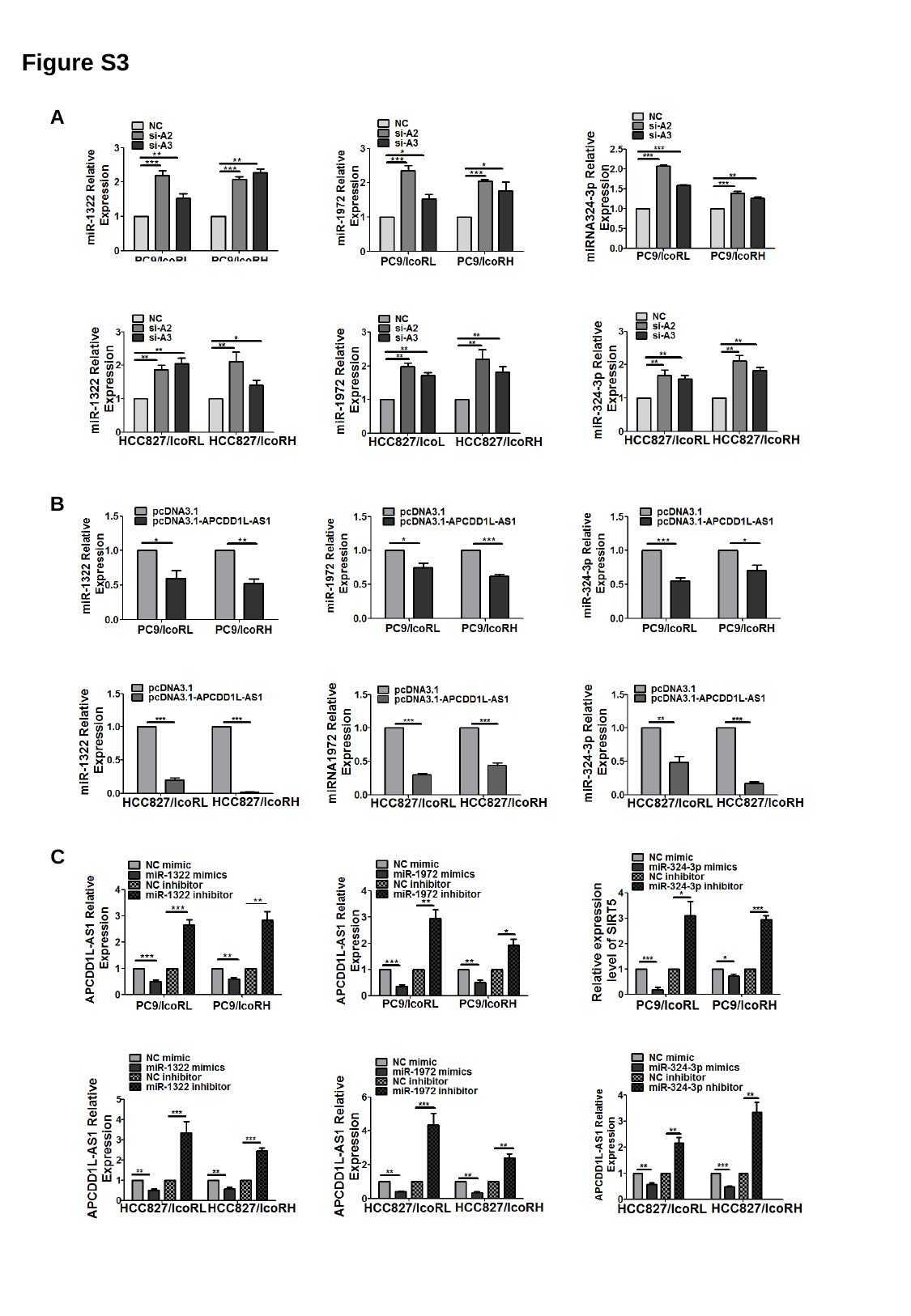

Figure S3
A
B
C

## Slide 5
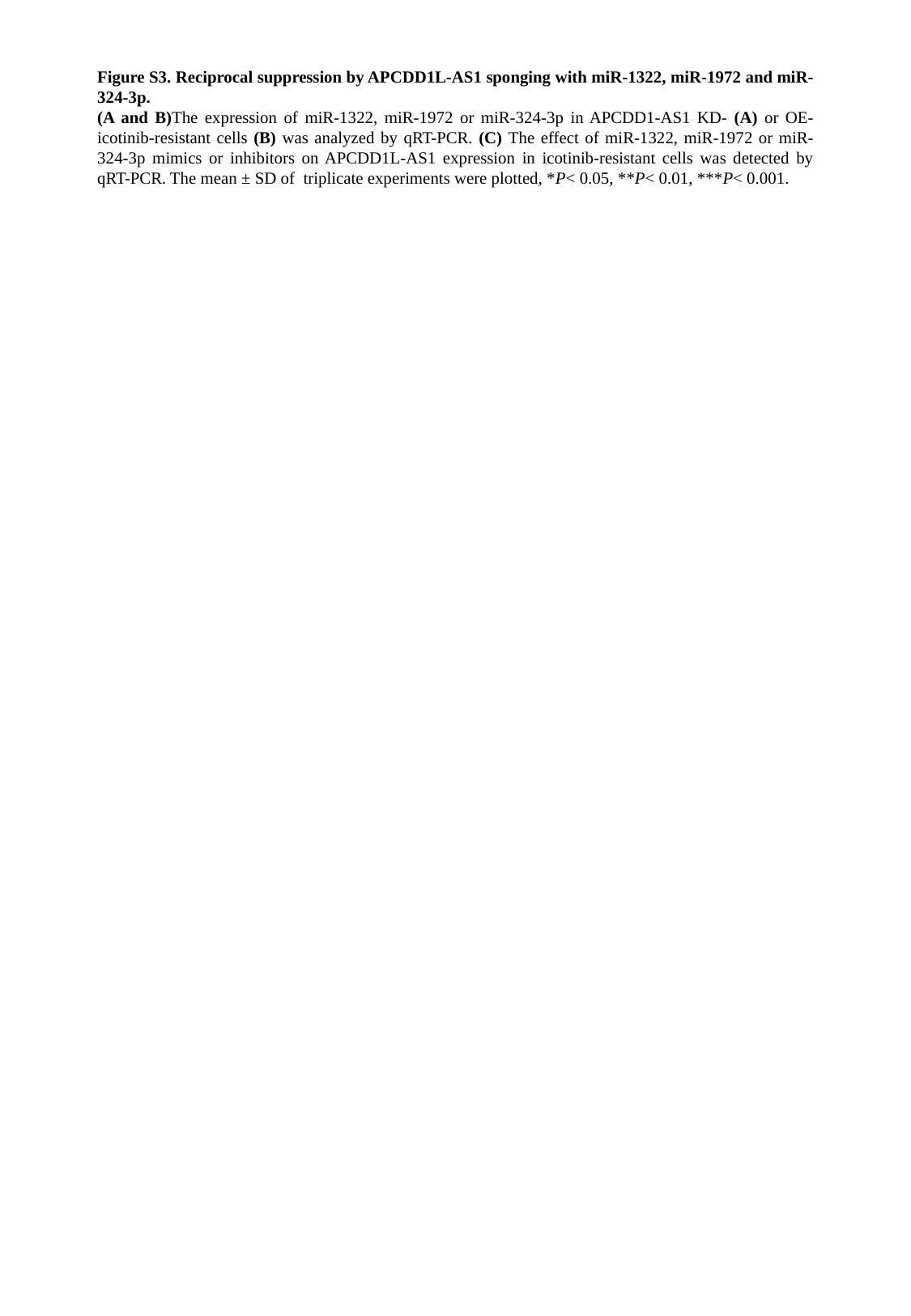

Figure S3. Reciprocal suppression by APCDD1L-AS1 sponging with miR-1322, miR-1972 and miR-324-3p.
(A and B)The expression of miR-1322, miR-1972 or miR-324-3p in APCDD1-AS1 KD- (A) or OE-icotinib-resistant cells (B) was analyzed by qRT-PCR. (C) The effect of miR-1322, miR-1972 or miR-324-3p mimics or inhibitors on APCDD1L-AS1 expression in icotinib-resistant cells was detected by qRT-PCR. The mean ± SD of triplicate experiments were plotted, *P< 0.05, **P< 0.01, ***P< 0.001.

## Slide 6
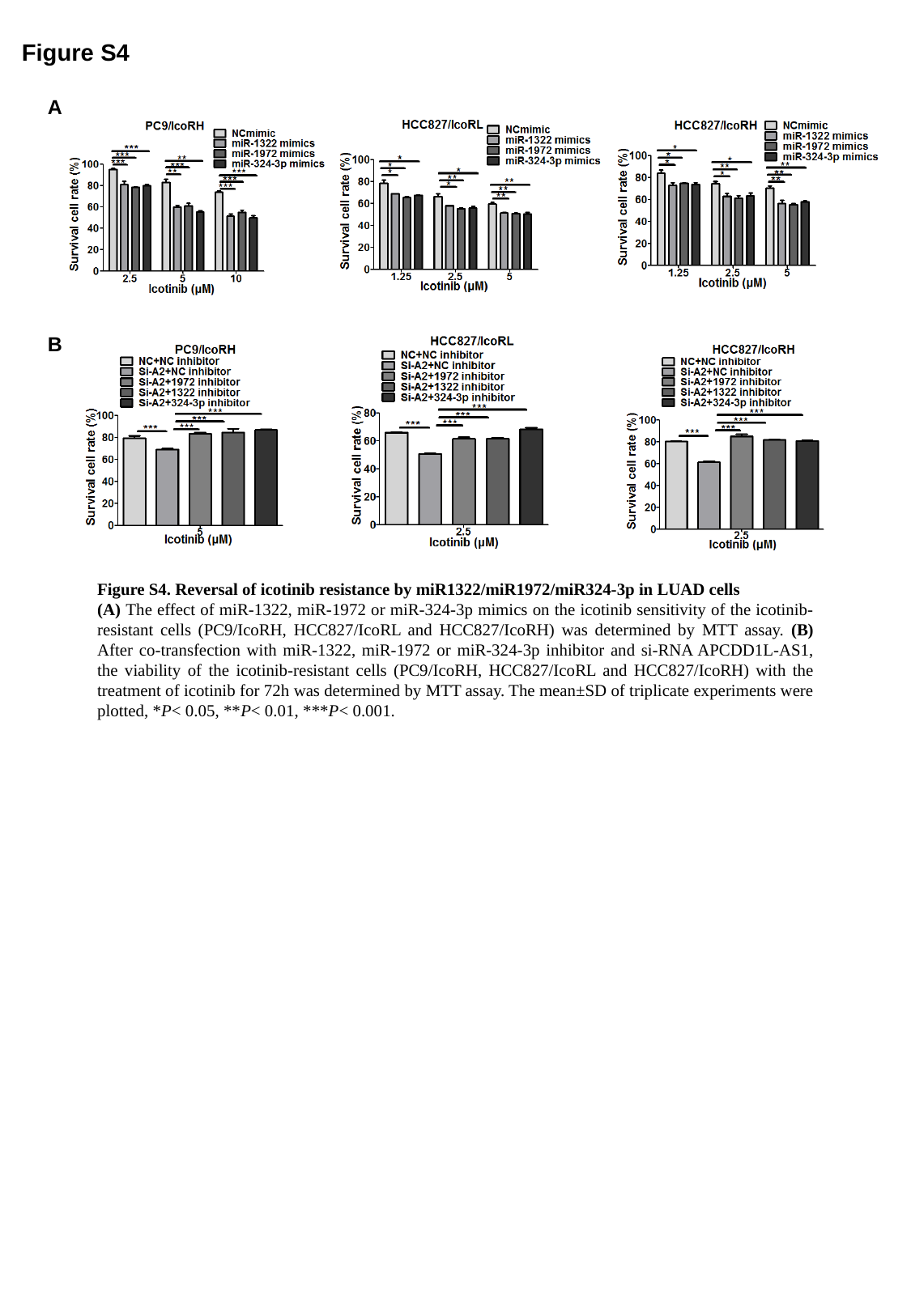

Figure S4
A
B
Figure S4. Reversal of icotinib resistance by miR1322/miR1972/miR324-3p in LUAD cells
(A) The effect of miR-1322, miR-1972 or miR-324-3p mimics on the icotinib sensitivity of the icotinib-resistant cells (PC9/IcoRH, HCC827/IcoRL and HCC827/IcoRH) was determined by MTT assay. (B) After co-transfection with miR-1322, miR-1972 or miR-324-3p inhibitor and si-RNA APCDD1L-AS1, the viability of the icotinib-resistant cells (PC9/IcoRH, HCC827/IcoRL and HCC827/IcoRH) with the treatment of icotinib for 72h was determined by MTT assay. The mean±SD of triplicate experiments were plotted, *P< 0.05, **P< 0.01, ***P< 0.001.

## Slide 7
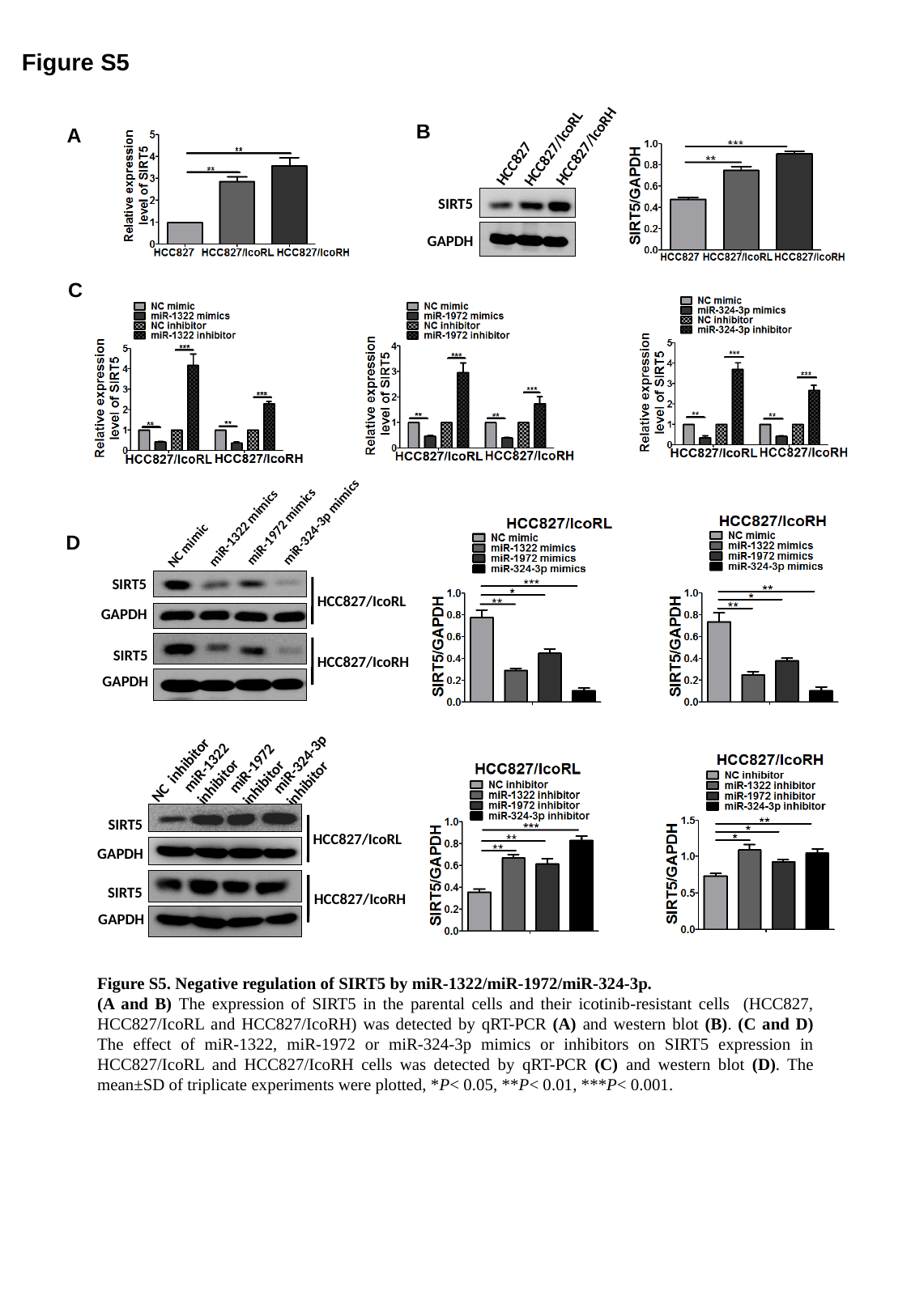

Figure S5
B
A
HCC827/IcoRH
HCC827/IcoRL
HCC827
SIRT5
GAPDH
C
miR-1972 mimics
miR-324-3p mimics
miR-1322 mimics
NC mimic
D
SIRT5
HCC827/IcoRL
GAPDH
SIRT5
HCC827/IcoRH
GAPDH
miR-1972 inhibitor
miR-324-3p inhibitor
miR-1322 inhibitor
NC inhibitor
SIRT5
HCC827/IcoRL
GAPDH
SIRT5
HCC827/IcoRH
GAPDH
Figure S5. Negative regulation of SIRT5 by miR-1322/miR-1972/miR-324-3p.
(A and B) The expression of SIRT5 in the parental cells and their icotinib-resistant cells (HCC827, HCC827/IcoRL and HCC827/IcoRH) was detected by qRT-PCR (A) and western blot (B). (C and D) The effect of miR-1322, miR-1972 or miR-324-3p mimics or inhibitors on SIRT5 expression in HCC827/IcoRL and HCC827/IcoRH cells was detected by qRT-PCR (C) and western blot (D). The mean±SD of triplicate experiments were plotted, *P< 0.05, **P< 0.01, ***P< 0.001.

## Slide 8
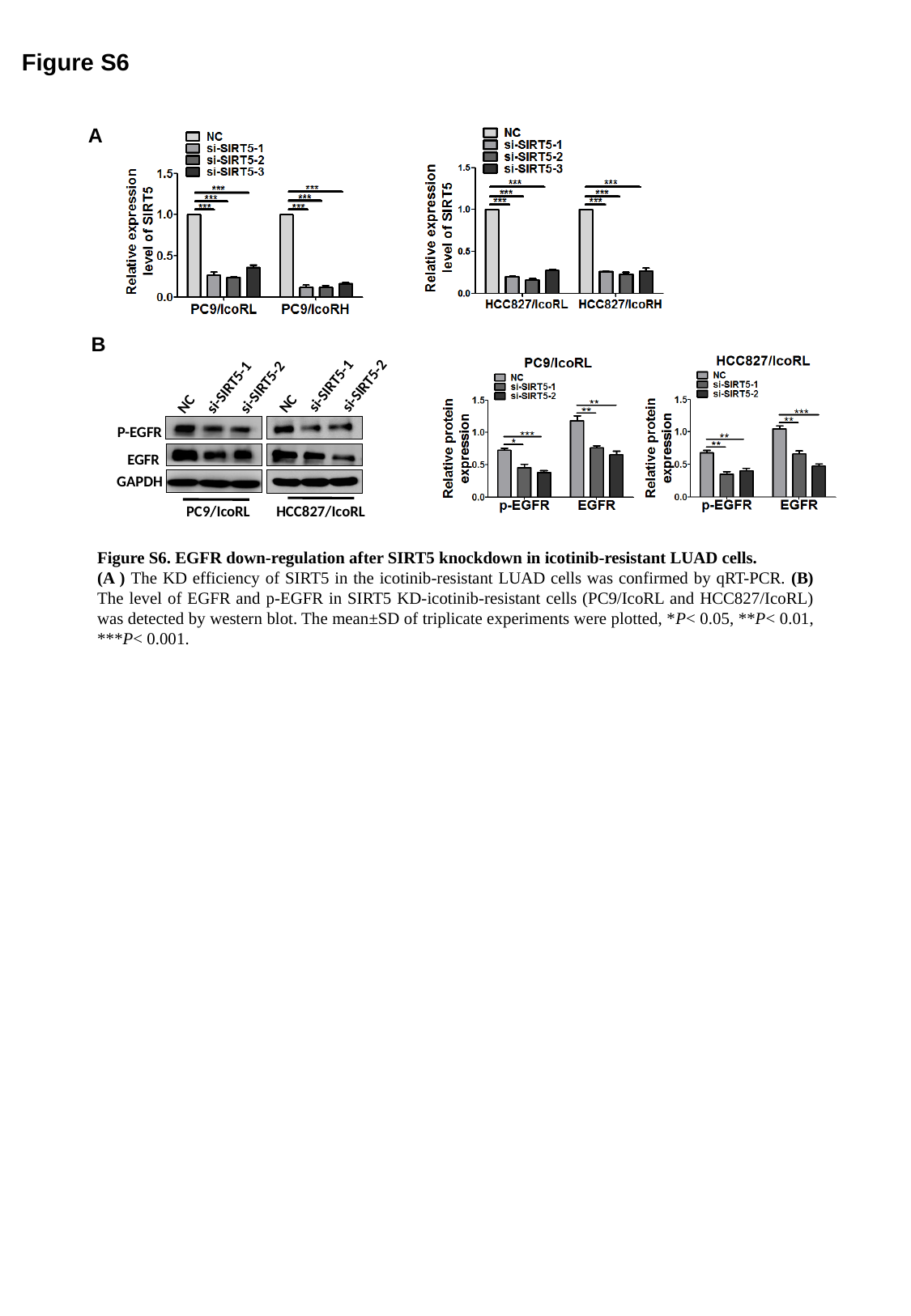

Figure S6
A
B
si-SIRT5-1
si-SIRT5-2
si-SIRT5-1
si-SIRT5-2
NC
NC
P-EGFR
EGFR
GAPDH
PC9/IcoRL
HCC827/IcoRL
Figure S6. EGFR down-regulation after SIRT5 knockdown in icotinib-resistant LUAD cells.
(A ) The KD efficiency of SIRT5 in the icotinib-resistant LUAD cells was confirmed by qRT-PCR. (B) The level of EGFR and p-EGFR in SIRT5 KD-icotinib-resistant cells (PC9/IcoRL and HCC827/IcoRL) was detected by western blot. The mean±SD of triplicate experiments were plotted, *P< 0.05, **P< 0.01, ***P< 0.001.

## Slide 9
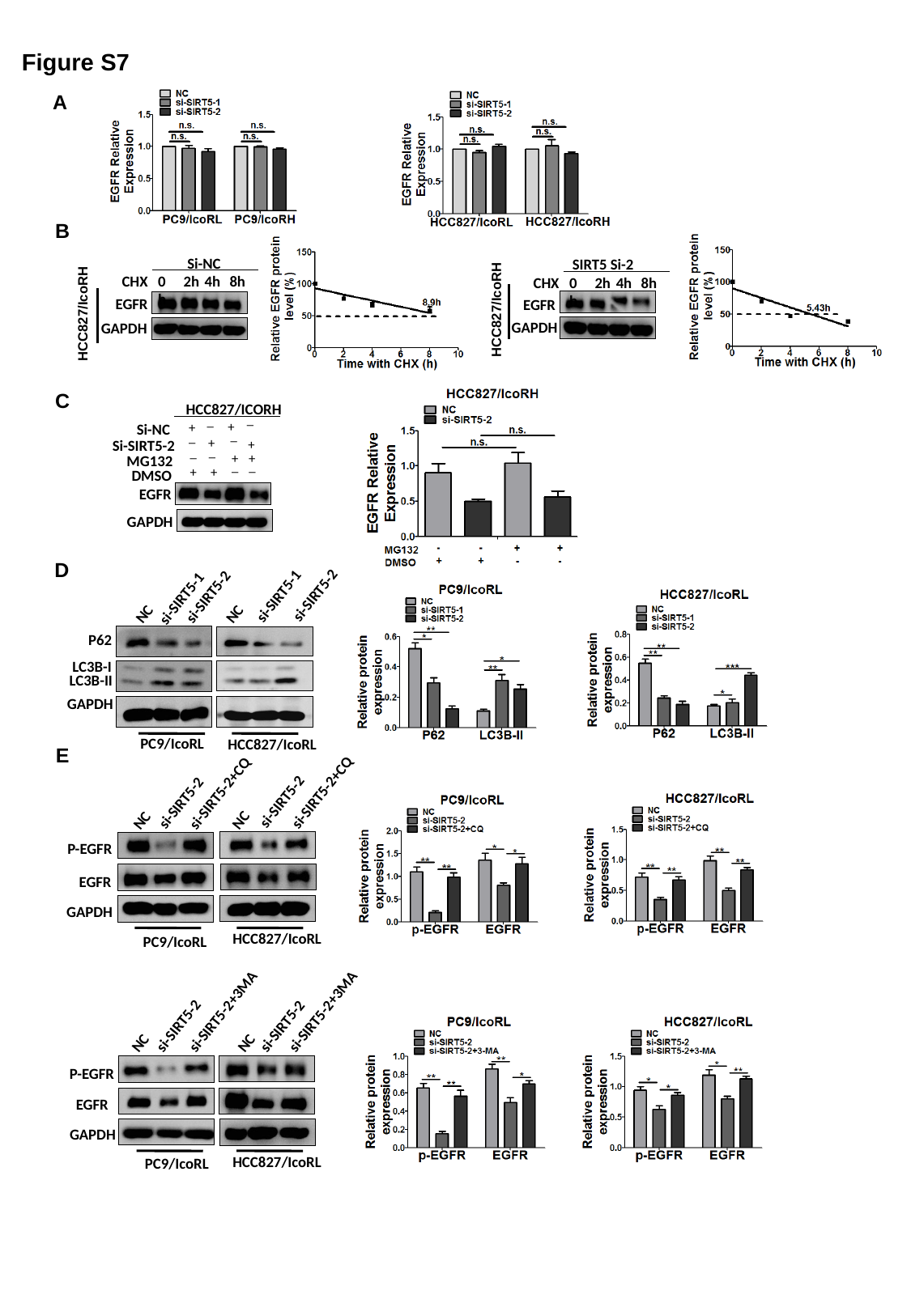

Figure S7
A
B
Si-NC
SIRT5 Si-2
CHX
0h
2h
4h
8h
CHX
0h
2h
4h
8h
EGFR
EGFR
HCC827/IcoRH
HCC827/IcoRH
GAPDH
GAPDH
C
HCC827/ICORH
_
_
+
+
Si-NC
_
_
+
+
Si-SIRT5-2
_
_
+
+
MG132
_
_
+
+
DMSO
EGFR
GAPDH
D
si-SIRT5-2
si-SIRT5-1
si-SIRT5-2
si-SIRT5-1
NC
NC
P62
LC3B-I
LC3B-II
 GAPDH
PC9/IcoRL
HCC827/IcoRL
E
si-SIRT5-2+CQ
si-SIRT5-2+CQ
si-SIRT5-2
si-SIRT5-2
NC
NC
P-EGFR
EGFR
GAPDH
HCC827/IcoRL
PC9/IcoRL
si-SIRT5-2+3MA
si-SIRT5-2+3MA
si-SIRT5-2
si-SIRT5-2
NC
NC
P-EGFR
EGFR
GAPDH
HCC827/IcoRL
PC9/IcoRL

## Slide 10
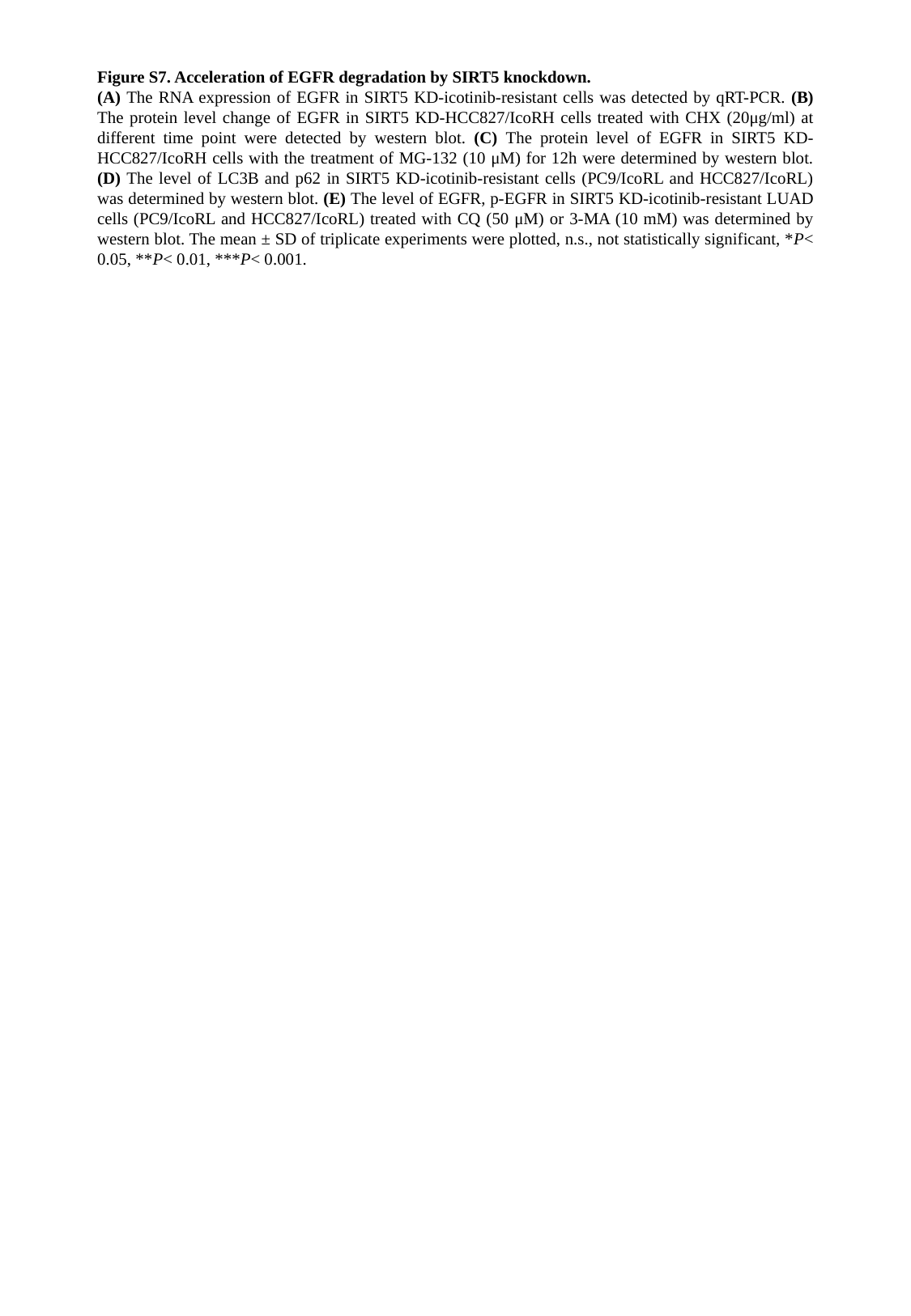

Figure S7. Acceleration of EGFR degradation by SIRT5 knockdown.
(A) The RNA expression of EGFR in SIRT5 KD-icotinib-resistant cells was detected by qRT-PCR. (B) The protein level change of EGFR in SIRT5 KD-HCC827/IcoRH cells treated with CHX (20μg/ml) at different time point were detected by western blot. (C) The protein level of EGFR in SIRT5 KD-HCC827/IcoRH cells with the treatment of MG-132 (10 μM) for 12h were determined by western blot. (D) The level of LC3B and p62 in SIRT5 KD-icotinib-resistant cells (PC9/IcoRL and HCC827/IcoRL) was determined by western blot. (E) The level of EGFR, p-EGFR in SIRT5 KD-icotinib-resistant LUAD cells (PC9/IcoRL and HCC827/IcoRL) treated with CQ (50 μM) or 3-MA (10 mM) was determined by western blot. The mean ± SD of triplicate experiments were plotted, n.s., not statistically significant, *P< 0.05, **P< 0.01, ***P< 0.001.

## Slide 11
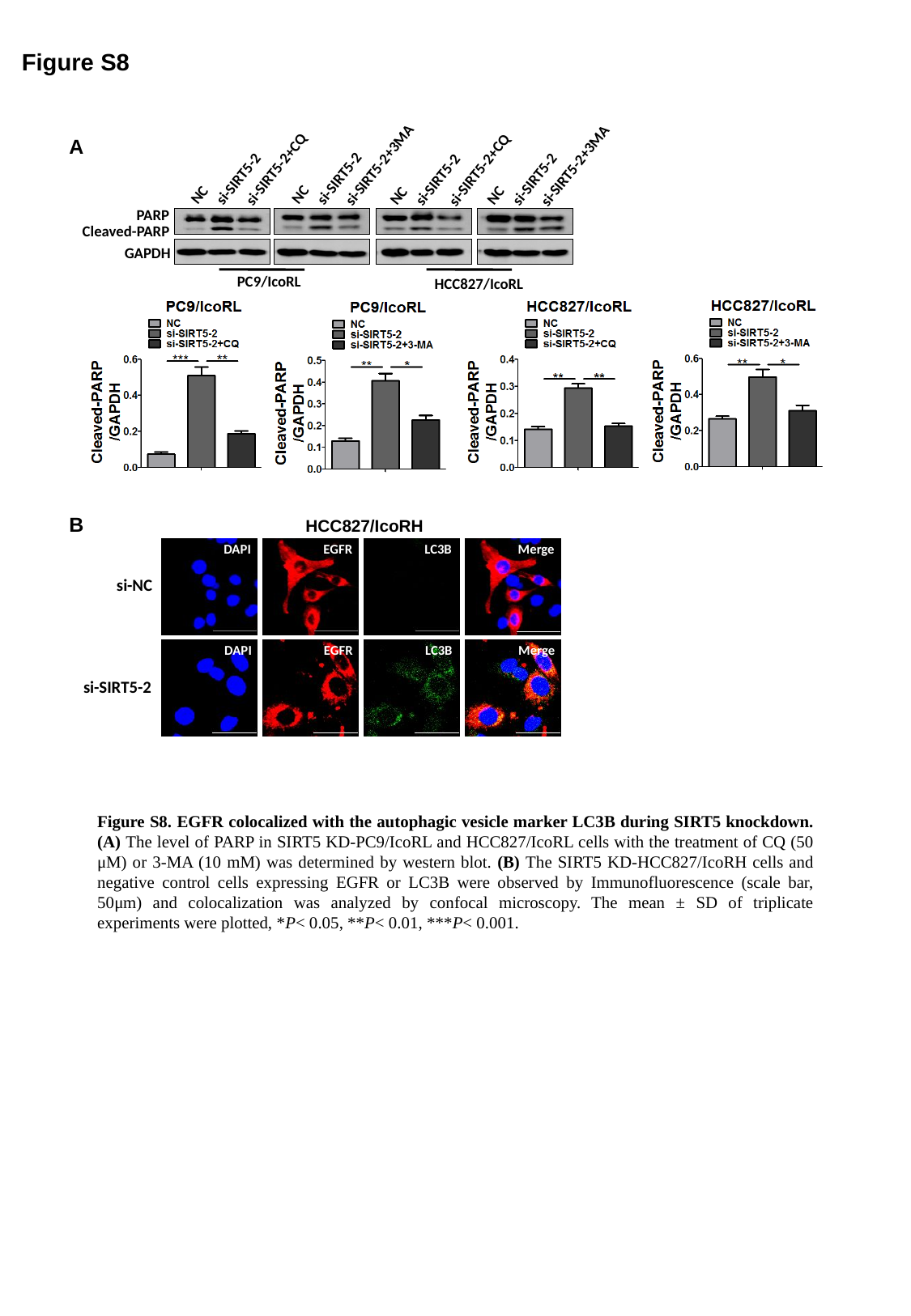

Figure S8
A
si-SIRT5-2+3MA
si-SIRT5-2+3MA
si-SIRT5-2+CQ
si-SIRT5-2+CQ
si-SIRT5-2
si-SIRT5-2
si-SIRT5-2
si-SIRT5-2
NC
NC
NC
NC
PARP
 Cleaved-PARP
GAPDH
PC9/IcoRL
HCC827/IcoRL
B
HCC827/IcoRH
DAPI
EGFR
LC3B
Merge
si-NC
DAPI
EGFR
LC3B
Merge
si-SIRT5-2
Figure S8. EGFR colocalized with the autophagic vesicle marker LC3B during SIRT5 knockdown. (A) The level of PARP in SIRT5 KD-PC9/IcoRL and HCC827/IcoRL cells with the treatment of CQ (50 μM) or 3-MA (10 mM) was determined by western blot. (B) The SIRT5 KD-HCC827/IcoRH cells and negative control cells expressing EGFR or LC3B were observed by Immunofluorescence (scale bar, 50μm) and colocalization was analyzed by confocal microscopy. The mean ± SD of triplicate experiments were plotted, *P< 0.05, **P< 0.01, ***P< 0.001.

## Slide 12
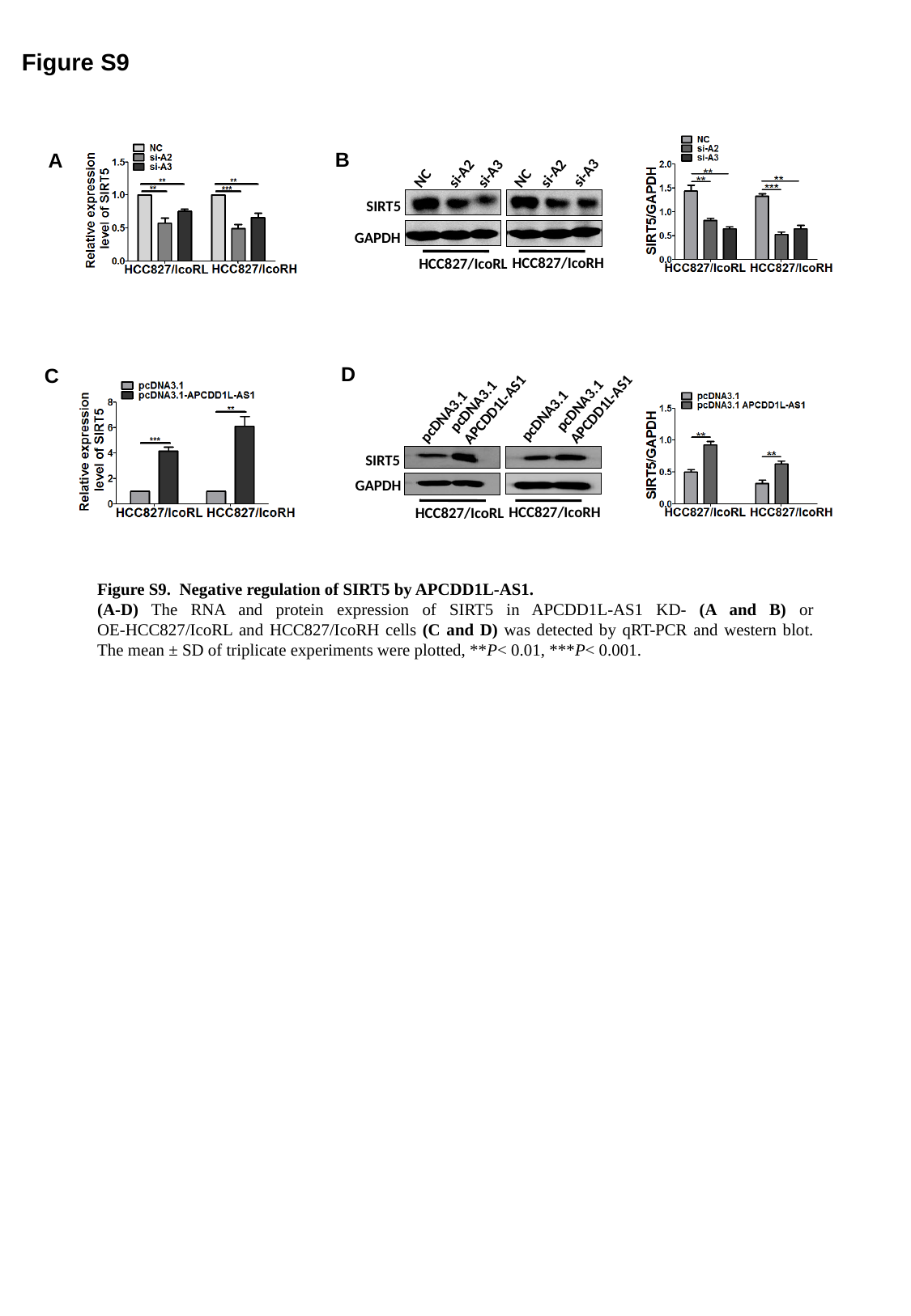

Figure S9
B
A
si-A3
si-A2
si-A2
si-A3
NC
NC
SIRT5
 GAPDH
HCC827/IcoRH
HCC827/IcoRL
D
C
pcDNA3.1
APCDD1L-AS1
pcDNA3.1
APCDD1L-AS1
pcDNA3.1
pcDNA3.1
SIRT5
 GAPDH
HCC827/IcoRH
HCC827/IcoRL
Figure S9. Negative regulation of SIRT5 by APCDD1L-AS1.
(A-D) The RNA and protein expression of SIRT5 in APCDD1L-AS1 KD- (A and B) or OE-HCC827/IcoRL and HCC827/IcoRH cells (C and D) was detected by qRT-PCR and western blot. The mean ± SD of triplicate experiments were plotted, **P< 0.01, ***P< 0.001.

## Slide 13
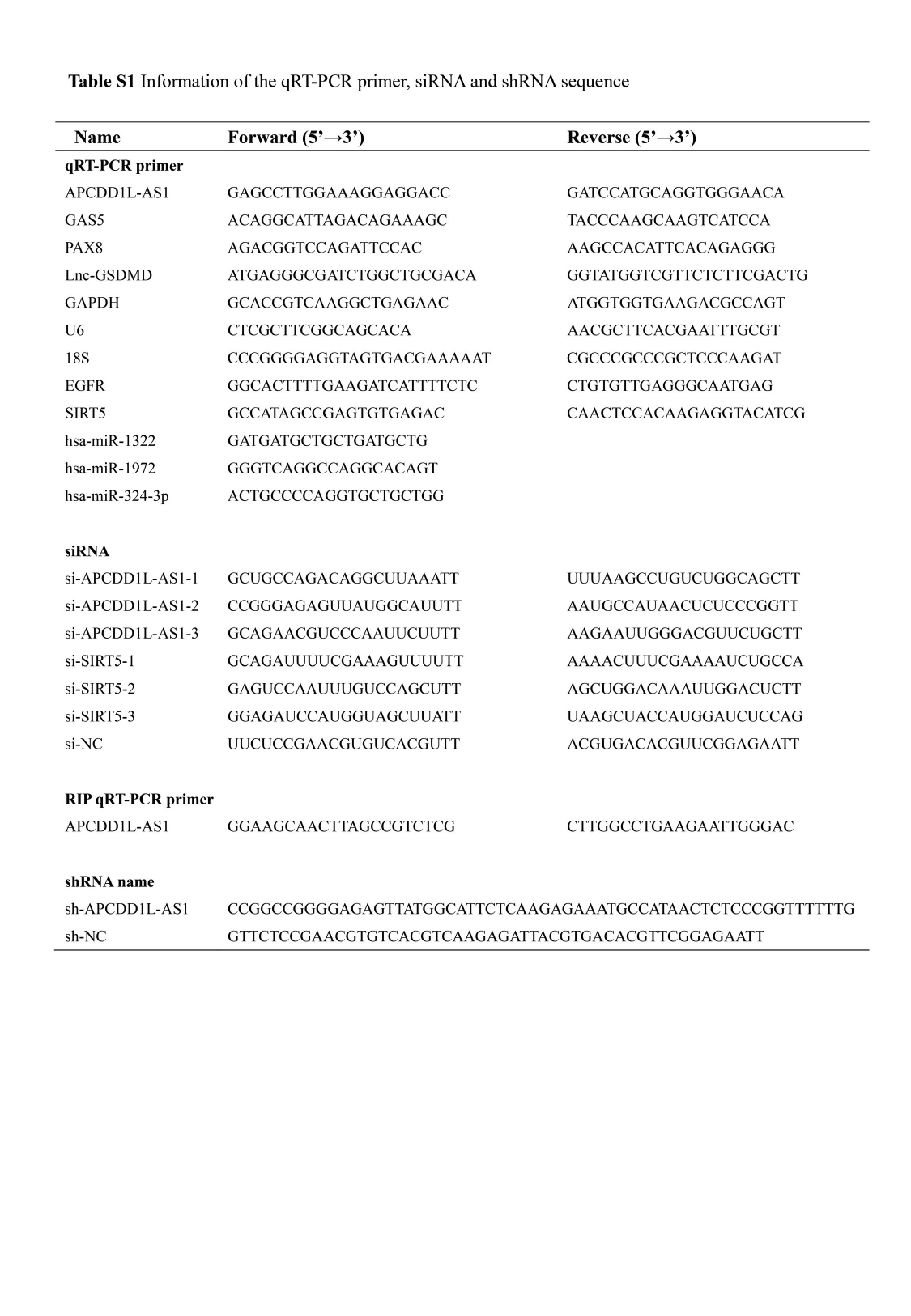

Supplement: Supplementary file 1 — Additional file 1: Figure S1. EGFR up-regulation and apoptosis inhibition by APCDD1L-AS1 in icotinib-resistant LUAD cells. Figure S2. Efficiency of miR-1322/miR-1972/miR-324-3p knockdown and overexpression. Figure S3. Reciprocal suppression by APCDD1L-AS1 sponging with miR-1322, miR-1972 and miR-324-3p. Figure S4. Reversal of icotinib resistance by miR1322/miR1972/miR324-3p in LUAD cells. Figure S5. Negative regulation of SIRT5 by miR-1322/miR-1972/miR-324-3p. Figure S6. EGFR down-regulation after SIRT5 knockdown in icotinib-resistant LUAD cells. Figure S7. Acceleration of EGFR degradation by SIRT5 knockdown. Figure S8. EGFR colocalized with the autophagic vesicle marker LC3B during SIRT5 knockdown. Figure S9. Negative regulation of SIRT5 by APCDD1L-AS1. Table S1. Information of the qRT-PCR primer, siRNA and shRNA sequence. [file 40364_2021_262_MOESM1_ESM.pptx]
